# Small extracellular vesicles in plasma reveal molecular effects of modified Mediterranean-ketogenic diet in participants with mild cognitive impairment

**DOI:** 10.1093/braincomms/fcac262

**Published:** 2022-10-19

**Authors:** Ashish Kumar, Mitu Sharma, Yixin Su, Sangeeta Singh, Fang-Chi Hsu, Bryan J Neth, Thomas C Register, Kaj Blennow, Henrik Zetterberg, Suzanne Craft, Gagan Deep

**Affiliations:** Department of Cancer Biology, Wake Forest School of Medicine, Winston-Salem, North Carolina 27157, USA; Department of Cancer Biology, Wake Forest School of Medicine, Winston-Salem, North Carolina 27157, USA; Department of Cancer Biology, Wake Forest School of Medicine, Winston-Salem, North Carolina 27157, USA; Department of Cancer Biology, Wake Forest School of Medicine, Winston-Salem, North Carolina 27157, USA; Department of Biostatistics and Data Science, Wake Forest School of Medicine, Winston-Salem, North Carolina 27101, USA; Wake Forest Baptist Comprehensive Cancer Center, Wake Forest School of Medicine, Winston-Salem, North Carolina 27157, USA; Department of Neurology, Mayo Clinic, Rochester, Minnesota 55905, USA; J Paul Sticht Center for Healthy Aging and Alzheimer’s Prevention, Wake Forest School of Medicine, Winston-Salem, North Carolina 27157, USA; Section on Comparative Medicine, Department of Pathology, Wake Forest School of Medicine, Winston-Salem, North Carolina 27157, USA; Department of Psychiatry and Neurochemistry, Institute of Neuroscience and Physiology, The Sahlgrenska Academy at the University of Gothenburg, S-431 80 Mölndal, Sweden; Clinical Neurochemistry Laboratory, Sahlgrenska University Hospital, S-431 80 Mölndal, Sweden; Department of Psychiatry and Neurochemistry, Institute of Neuroscience and Physiology, The Sahlgrenska Academy at the University of Gothenburg, S-431 80 Mölndal, Sweden; Clinical Neurochemistry Laboratory, Sahlgrenska University Hospital, S-431 80 Mölndal, Sweden; Department of Neurodegenerative Disease, UCL Queen Square Institute of Neurology, Queen Square, WC1E6AE London, UK; UK Dementia Research Institute at UCL, WC1E6AE London, UK; Department of Internal Medicine-Gerontology and Geriatric Medicine, Wake Forest School of Medicine, Winston-Salem, North Carolina 27157, USA; Department of Cancer Biology, Wake Forest School of Medicine, Winston-Salem, North Carolina 27157, USA; Wake Forest Baptist Comprehensive Cancer Center, Wake Forest School of Medicine, Winston-Salem, North Carolina 27157, USA; J Paul Sticht Center for Healthy Aging and Alzheimer’s Prevention, Wake Forest School of Medicine, Winston-Salem, North Carolina 27157, USA

**Keywords:** extracellular vesicles, mild cognitive impairment, ketogenic diet, amyloid β, glutamate receptor

## Abstract

Extracellular vesicles have emerged as a less-invasive nano-tool for discovering biomarkers of Alzheimer’s disease and related dementia. Here, we analysed different neuron-enriched extracellular vesicles from plasma to predict response and molecular mechanisms of ketogenic diet’s efficacy in mild cognitive impairment participants. The study was a randomized crossover design in which cognitively normal and mild cognitive impairment participants consumed a modified Mediterranean-ketogenic diet or American Heart Association diet for 6 weeks, followed by other diet after washout. L1 cell adhesion molecule, synaptophysin and neural cell adhesion molecule surface markers were used to enrich for neuron-secreted small extracellular vesicles (sEV^L1CAM^, sEV^SYP^ and sEV^NCAM^). For the first time, we have presented multiple evidences, including immunogold labelling/transmission electron microscopy, clusters of differentiation 63-ELISA-based assay, confocal microscopy fluorescent images and flow cytometry data confirming the presence of L1 cell adhesion molecule on the surface of sEV^L1CAM^, validating purity and relative abundance of sEV^L1CAM^ in the plasma. Cargo analysis of sEV^L1CAM^ showed that modified Mediterranean-ketogenic diet intervention reduces amyloid beta 1–42 (50.3%, *P* = 0.011), p181-tau (34.9%, *P* = 0.033) and neurofilament light (54.2%, *P* = 0.020) in mild cognitive impairment participants. Moreover, sEV^L1CAM^showed better sensitivity compared with CSF in analysing increased glutamate (6-folds, *P* < 0.0001) from mild cognitive impairment participants following modified Mediterranean-ketogenic diet intervention. sEV^L1CAM^ characterization also suggested that modified Mediterranean-ketogenic diet differentially targets the expression of various glutamate receptors—glutamate receptor ionotropic NMDA1, glutamate receptor ionotropic NMDA2A, glutamate receptor ionotropic NMDA2B and glutamate receptor ionotropic AMPA type subunit 1. Importantly, these sEV^L1CAM^ measures strongly correlated with corresponding clinical CSF biomarkers (neurogranin, amyloid beta 1–42, neurofilament light and tau). Furthermore, sEV^L1CAM^ were loaded with less advanced glycation endproducts and exhibited anti-inflammatory activity following modified Mediterranean-ketogenic diet intervention. Most importantly, the expression of monocarboxylate transporter 2 on the surface of sEV^L1CAM^ predicted the amyloid beta 1–42 response to modified Mediterranean-ketogenic diet intervention (area under the curve = 0.87, *P* = 0.0044) and offered a novel screening tool to identify participants responsive to this dietary intervention. Finally, sEV^L1CAM^, sEV^SYP^ and sEV^NCAM^ showed significantly high concordance in analysing amyloid beta 1–42 (Pearson correlation coefficient ≥ 0.63, *P* < 0.01) and neurofilament light (Pearson correlation coefficient ≥ 0.49, *P* < 0.05). Together, small extracellular vesicles in plasma offers promise in assessing the efficacy of dietary/therapeutic intervention against mild cognitive impairment/Alzheimer’s disease.

## Teaser

Kumar *et al*. analysed the neuronal-enriched small extracellular vesicles (NEE) from plasma to demonstrate the effect of modified Mediterranean-ketogenic diet in reducing Alzheimer’s disease-related biomarkers, altering glutamate and glutamate receptors and reducing inflammatory response. The present study supports the usefulness of NEE in developing novel preventive/therapeutic interventions for Alzheimer’s disease.

## Graphical Abstract

**Graphical abstract:**
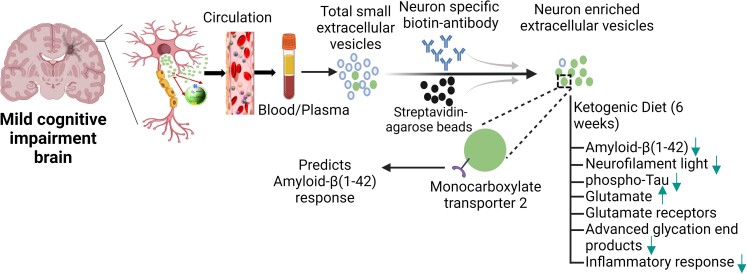

## Introduction

Alzheimer’s disease is a fatal neurodegenerative disorder with limited availability of proven disease-modifying treatment or preventative intervention.^1^ One emerging unconventional approach is the dietary intervention based upon ketogenic diet (KD), i.e. a low carbohydrate, adequate-protein and high-fat diet that leads to increased liver production of ketone bodies (i.e. β-hydroxybutyrate and aceto-acetate) that are readily transported into the brain causing a shift from glucose to ketone bodies as the primary energy source.^2^ Though the precise mechanisms underlying the effectiveness of the KD are not fully elucidated, candidates like reduction of neuronal hyperexcitability through glutamatergic and amyloid-beta (Aβ) inhibition and KD-mediated reduction of oxidative stress, advanced glycation endproducts (AGEs) and neuroinflammation have been suggested to play a critical role.^3–8^ For example, in Alzheimer’s disease, Aβ oligomers interfere with glutamate receptors in the synapses and increase the spillover of glutamate, activating glutamate receptors in the extra-synaptic sites, which in turn activate apoptotic and necrotic pathways.^9,10^ Preclinical studies provide ample evidence that elevating ketone bodies regulate Aβ levels.^11–13^ The 3xTgAD mice treated with a ketone-inducing intervention showed less amyloid and/or tau pathology and improved memory performance.^11,12^ Ketones reduce Aβ neurotoxicity by blocking its entry into neurons and decreasing amyloid aggregation, with associated improvement in memory.^13^

Monocarboxylate transporters (MCTs) are responsible for the transport of pyruvate, lactate as well as ketone bodies across the blood–brain barrier. An MCT subtype, MCT2, has a higher affinity for ketone substrate compared with MCT1 and 4 and is expressed at a higher level in cells where rapid uptake is required at low substrate concentration.^14^ Several studies have examined the effects of elevating ketones with medium-chain triglyceride supplements in Alzheimer’s disease.^3,15,16^ Recently, we reported the results of a pilot clinical study where we compared the effect of a high-fat and low-carbohydrate modified Mediterranean-ketogenic diet (MMKD) and a low-fat American Heart Association diet (AHAD) on 11 cognitively normal (CN) older adults and 9 adults with amnestic mild cognitive impairment (MCI).^17^ Outcomes showed that MMKD was well-tolerated and associated with increased CSF Aβ1–42 and decreased tau levels.^17^ Also, there was increased cerebral perfusion and increased cerebral ketone body uptake accessed by ^11^C-acetoacetate PET following MMKD intervention.^17^ Several studies have also suggested that KD targets multiple pathways to inhibit inflammation.^18,19^ These studies suggest a potential therapeutic role of the KD in Alzheimer’s disease but are limited in identifying potential molecular targets and mechanisms of action of beneficial effect. Moreover, the molecular effects of the KD diet are not well defined in humans, mainly due to a lack of access to brain tissue. The identification of molecular mechanisms underlying KD diet efficacy would be helpful in establishing this approach as an ‘evidence-based’ intervention against Alzheimer’s disease. Extracellular vesicles (EV) in the peripheral blood could be potentially useful in understanding the molecular and pathophysiological state of neuronal cells.

EV are lipid-bound vesicles secreted by cells into the extracellular space, which play a key role in intercellular communication and maintenance of cellular homeostasis. EV are quite heterogeneous and can be subcategorized based upon their biogenesis and release pathway, size, content and functions. Exosomes (∼30–150 nm) are small EV (sEV) of endocytic origin, while microvesicles (100 nm ≥ 1 μm) bud directly from the plasma membrane. Isolation of cell type-specific EV from plasma has garnered much attention in accessing the pathophysiological state of the ‘difficult to access’ cells/tissues. Recently, the discovery of neuronal EV in plasma has led to studies examining their role as ‘liquid biopsies’ for Alzheimer’s disease and related dementias (ADRD).^20–29^ For example, Fiandaca *et al*. reported that a combination of p181-tau, p-S396-tau and Aβ1–42 contained within neuronal EV could predict the development of Alzheimer’s disease up to 10 years before the clinical onset.^28^ Another study showed that abnormal plasma neuronal EV levels of p-tau, Aβ1–42, neurogranin (Ng) and repressor element 1-silencing transcription factor accurately predicted the conversion of MCI to Alzheimer’s disease.^21^ Similarly, auto-lysosomal protein levels,^27^ transcription factors^30^ and phosphorylated forms of insulin receptor substrate^20^ in neuronal EV have also correctly distinguished 100% of participants with Alzheimer’s disease from normal controls. Recently, neuronal EV isolated from plasma have also shown the potential of identifying the efficacy of an antidepressant drug on major depressive disorder subjects.^31^ Though L1CAM has been extensively used for the isolation/enrichment of neuronal EV, the specificity, validity and even its presence on sEV surface have been questioned.^32^ Other concerns like the amount of surface biomarkers on neuronal EV in plasma, the percentage of neuronal EV in the blood, their CNS origin, and most importantly, the potential of neuronal EV to assess the treatment response of conventional/interventional therapies still need to be addressed.

In the present manuscript, utilizing archived plasma from the above-mentioned pilot clinical study,^17^ we isolated and characterized neuronal-enriched sEV (NEE) using different surface markers like L1CAM, synaptophysin (SYP) and neural cell adhesion molecule (NCAM), denoted as sEV^L1CAM^, sEV^SYP^ and sEV^NCAM^, respectively. We analysed the levels of various ADRD markers i.e. Aβ1–42, total-tau, p181-tau and neurofilament light (NfL), in sEV^L1CAM^before and after MMKD and AHAD intervention. To understand the potential molecular effects of these dietary interventions, we also assessed the expression of different glutamate receptors, glutamate, AGEs and pro-inflammatory effect of sEV^L1CAM^ in an *ex vivo* assay. Lastly, we validated a few of the results observed in sEV^L1CAM^ (such as Aβ1–42 and NfL expression) in NEE isolated using other surface markers (synaptophysin and NCAM). Results demonstrate NEE utility in understanding the molecular effects underlying the efficacy of MMKD against amnestic MCI. NEE analyses suggested a pleiotropic molecular mechanism of action of MMKD through targeting Aβ-glutamate-glutamate receptor signalling leading to reduced inflammation and neurodegeneration. We also identified surface expression of MCT2 on sEV^L1CAM^ useful in potentially distinguishing ‘responders’ versus ‘non-responders’ to MMKD intervention.

## Materials and Methods

### Plasma and CSF samples

Archived plasma and CSF samples were obtained from a recently published study.^17^ Briefly, in the completed study (approved by the Wake Forest Institutional Review Board; ClinicalTrials.gov Identifier: NCT02984540), participants were divided into two cognitive subgroups: CN adults with subjective memory complaints diagnosed using Alzheimer’s disease Neuroimaging Initiative criteria; and adults with amnestic MCI diagnosed by expert physicians and neuropsychologists using the National Institute on Aging at National Institutes of Health and the Alzheimer's Association guidelines. Major inclusion and exclusion criteria for participant’s recruitment have been reported earlier.^17^ The demographic details of participants are provided in Supplementary Table 1. The study consisted of a randomized crossover design in which participants (both CN and amnestic MCI) consumed either MMKD or the control AHAD for 6 weeks, followed by a 6-week washout period in which participants were instructed to resume their pre-study diet, after which the second diet was consumed for 6 weeks. The proportions of carbohydrates and fat were the main variables manipulated between the two diets. The target macronutrient composition (expressed as % of total calories) was 5–10% carbohydrate, 60–65% fat and 30% protein for MMKD; and 55–65% carbohydrate, 15–20% fat and 20–30% protein for AHAD. Prior to diet randomization, baseline characterization of cognitive status, lumbar puncture (LP), MRI and metabolic profiles were performed. Cognitive function, LP, MRI and metabolic parameters were reassessed after each diet. The fasting blood was collected before and after each diet. Blood samples were immediately placed on ice and spun within 30 min at 2200 rpm in a cold centrifuge for 15 min. The plasma was aliquoted into separate storage tubes and flash-frozen at −80°C until analysed. All assays were performed following the one-time thaw of frozen samples. Participants completed LP after a 12 h fast at baseline and after each diet for collection of CSF.

## Isolation of total EV (TE) from plasma and NEE isolation from TE

TE and NEE were isolated, as reported by us recently.^33^ Schematic representation of experimental steps is shown in Supplementary Fig. 1A. Briefly, plasma samples from CN (*n* = 11) and MCI (*n* = 9) from all pre- and post-diet conditions were centrifuged at 500 g for 5 min, 2000 g for 10 min followed by 10 000 g for 30 min at 4°C to remove the larger sized vesicles. The collected supernatant was treated with thromboplastin-D, and EV were isolated using the ExoQuick (System Biosciences, CA, USA) as reported earlier.^34^ For sEV^L1CAM^ isolation, TE were incubated overnight with biotin-tagged L1CAM antibody (Clone eBio5G3 [5G3], ThermoFisher Scientific, MA, USA). Next, streptavidin-tagged agarose resin (ThermoFisher, MA, USA) was added. Following incubation, EV bound to agarose resins were centrifuged, and the supernatant containing unbound EV were removed. Finally, sEV^L1CAM^ were removed from beads by adding IgG elution buffer (ThermoFisher, MA, USA), and pH of the eluate was neutralized by 1 M Tris base (pH = 9). Similarly, sEV^SYP^and sEV^NCAM^ were isolated using respective biotin-labelled antibodies (synaptophysin-biotin antibody from Novus Biologicals, CO, USA; and NCAM-biotin antibody from ThermoFisher, MA, USA). Human IgG isotype (biotin) was also incubated with TE as a control to repudiate the non-specific isolation of NEE/EV, following the same protocol and experimental conditions.

### Nanoparticle tracking analysis

Quantification of the hydrodynamic diameter distribution and concentration of EV were performed using the Nanosight NS300 (Malvern Instruments, UK) as reported by us recently.^33^ The instrument was primed with PBS (filtered through a 0.22 μm filter) and maintained at 25°C. Accurate nanoparticle tracking was verified using 100 and 200 nm polystyrene nanoparticle standards (Malvern Instruments) prior to capturing the samples. TE and NEE were diluted in 0.22 μm filtered PBS to measure size and concentration. Five measurements (30 s each) were obtained for each sample and their average was plotted.

### Immunogold labelling and transmission electron microscopy

For immunogold labelling, sEV^L1CAM^ were fixed with 2% paraformaldehyde in PBS buffer (pH 7.4), then adsorbed for 1 h to a carbon-coated grid. Clusters of differentiation 63 (CD63) antibody was conjugated with gold particles (20 nm) using a gold conjugation kit as per the manufacturer’s recommendation (Abcam, MA, USA). Samples were first incubated with primary antibody (L1CAM, CD63 and CD9) and then secondary antibody tagged with 10 nm gold particles or directly with anti-CD63–20 nm gold particles. sEV^L1CAM^ were stained with 1% uranyl acetate for 5 min, and images were captured on Tecnai T12 transmission electron microscopy (TEM).

### Exo-check antibody array

TE (50 µg) and sEV^L1CAM^ (20 µg) lysates were characterized for exosomal biomarkers using Exo-check exosome antibody array and Exo-check exosome antibody (neuro) array (outline presented in Supplementary Fig. 1B, left panel) (System Biosciences, Palo Alto, CA, USA), respectively, following vendor’s protocol.

### Co-localization of L1CAM and CD63

Intact NEE (sEV^L1CAM^ and sEV^NCAM^) were immobilized on a CD63 antibody-coated ELISA plate (RayBiotech, GA, USA) and washed thoroughly to remove any unbound NEE/EV or free protein. Next, NEE/EV bound to the surface of the plate were labelled with L1CAM-biotin or CD63-biotin antibody and then with streptavidin-HRP solution. Finally, one step 3,3′,5,5′-tetramethylbenzidine solution was added, and the plate was read at 450 nm (experimental plan presented in Supplementary Fig. 1C).

### Confocal microscopy

To confirm the purity of isolated sEV^L1CAM^ and also to confirm the presence of L1CAM on NEE surface (and co-expression with CD63), sEV^L1CAM^ were isolated as described above. Agarose resin bound sEV^L1CAM^ were incubated with L1CAM-PE (BioLegend, CA, USA) antibody for 1 h at RT in dark followed by incubation with 1 × membrane labelling dye CellBrite 488 (Biotium, CA, USA) for 15 min at RT. Agarose resin were washed three times and resuspended in 25 µl of filtered PBS. Agarose resin bound with sEV^L1CAM^ were then transferred on a clean microscopic slide, and coverslip was placed over it. Agarose resin tagged with IgG-biotin antibody was also used following similar experimental conditions and used as control. Slides were then imaged on Olympus FV1200 spectral laser scanning confocal microscope with 20 × or 40 × objective lens, with membrane labelling dye on green channel and L1CAM-PE with red pseudo-colour. Similarly, after isolating sEV^L1CAM^ with agarose resin, L1CAM-PE and CD63-APC antibodies were used to label agarose resin-bound sEV^L1CAM^ for 1 h at RT to analyse the co-expression of L1CAM and CD63 on sEV^L1CAM^ surface. After three washes, 25 µl of resin was transferred on a glass slide and imaged using pseudo green colour for PE signal (for L1CAM). Agarose resin tagged with IgG-biotin antibody, incubated with TE, were also labelled with L1CAM-PE and CD63-APC and used as control.

### Flow cytometry

To analyse the percentage of sEV^L1CAM^ in TE from CN and MCI participants, TE were labelled with membrane labelling dye CellBrite 488 (Biotium, CA, USA) with and without the L1CAM-PE (BioLegend, CA, USA) or synaptophysin-Alexa Fluor 647 (Novus Biologicals, CO, USA) antibody. TE without dye were used as control to set the gate for positively (dye) labelled EV (Supplementary Fig. 2A). TE labelled with dye but without L1CAM-PE/synaptophysin-AF647 antibody were used to set the gate for PE/AF647 positive events (Supplementary Fig. 2B). L1CAM/synaptophysin antibody and dye at the same dilution in PBS (filtered through 0.22 μm filter) were also analysed. A threshold cut of 2000 at violet side scatter was set up to exclude the machine background noise. PE and AF647 labelled isotype control were used to confirm the specificity of the fluorescence signals (Supplementary Fig. 2C). Samples were diluted 1:100 in filtered PBS before acquisition to achieve an abort ratio of less than 10%. All samples were acquired on CytoFlex (Beckman Coulter Life Science, Indianapolis, IN, USA) for 60 s at a low flow rate. Filtered PBS was run for 60 s in between the samples.

To confirm the dye positive events are EV and to negate the swarm effect, serial dilutions of samples were assessed. The linear reduction in total events with dilution in the gated regions confirmed that the positive events were EV (Supplementary Fig. 2D). L1CAM-PE and synaptophysin-AF647 antibody labelled EV were also serially diluted and measured for change in mean fluorescent intensity (Supplementary Fig. 2E). For further confirmation, 0.25% triton X-100 was added to the EV, and lysed samples were acquired. Gate applied to detect dye positive EV were applied to all the other samples to confirm the capture of EV only. Similarly, TE were analysed to identify the percentage of sEV^SYP^ (Supplementary Fig. 2F) using a synaptophysin-AF647 antibody.

A similar experimental set-up was used to characterize the NEE (Supplementary Fig. 3). The purity of isolated NEE was confirmed by three different surface markers; L1CAM, synaptophysin and neuron-specific enolase (ENO2). NEE (sEV^L1CAM^, sEV^SYP^ and sEV^NCAM^) were labelled with L1CAM-PE, synaptophysin-AF647 or ENO2-PE antibody at room temperature for 2 h. Thereafter, CellBrite dye at a final 1 × concentration (in filtered PBS) was used to label the NEE for 15 min at RT. NEE without dye were used to separate the NEE from background noise. Also, NEE with dye but without antibodies were used to set the gate for L1CAM-PE, ENO2-PE and synaptophysin-AF647 positive events. NEE samples were acquired for 60 s, with filtered PBS for 60 s in between the samples.

To measure the surface expression of MCT1 and 2, sEV^L1CAM^ were isolated using a biotin-labelled L1CAM antibody tagged on streptavidin-coated magnetic beads using our previously described method.^33^ Magnetic beads were washed four times, and sEV^L1CAM^ bound to magnetic beads were labelled with fluorescently tagged (AF647) antibodies for MCT1 and 2. Magnetic beads bound sEV^L1CAM^ were analysed by flow cytometry by acquiring total of 10 000 events (beads). Mean fluorescent intensities were calculated by FCS Express 7 software.

### ELISA and colorimetric assays

sEV^L1CAM^ were lysed by adding 10 × RIPA buffer (Milipore, Burlington, MA, USA, Cat no. 20-188) to a final concentration of 1×, and protein concentration of the lysate was quantified by the BCA method. sEV^L1CAM^ lysate was used for the analysis of Aβ1–42 (R&D Systems, Minneapolis, MN, USA), NfL (Abbexa, Houston, TX, USA), p-Tau (pT181), total Tau (both from Invitrogen, Carlsbad, CA, USA), GRIA1A, GRIA1B (both from MyBioSource, CA, USA), glutamate receptor ionotropic NMDA1 (GRIN1), glutamate receptor ionotropic NMDA2A (GRIN2A) (both from Novus Biologicals, CO, USA) and AGEs (MyBioSource, CA, USA) as per the manufacturers’ instructions. The concentration of glutamate in sEV^L1CAM^ and CSF was analysed using a bioluminescent assay (Glutamate-Glo assay, Promega, WI, USA). sEV^L1CAM^ lysate was used directly to estimate glutamate concentration as per the manufacturer’s recommendation.

### NF-κB activation assay

NF-κB activation assay was performed on THP-1 lucia NF-κB monocyte cells (InvivoGen, San Diego, CA, USA) as reported by us recently^33^ by treating cells with 10 µg of sEV^L1CAM^ for 16–18 h. Then, the activity of secreted luciferase was detected using QUANTi-Luc Gold, luminescence detection reagent (InvivoGen, San Diego, CA, USA).

### Statistical analysis

Data were analysed using GraphPad Prism 7.0 software (La Jolla, CA, USA) and SAS 9.4 software (SAS Inc., Cary, NC, USA). The distributions of the outcome measures were checked to ensure that the conditional normality assumption was satisfied. The comparisons of size, concentration and protein concentration per EV in TE and sEV^L1CAM^ between pre- and post-MMKD and AHAD conditions were performed using the paired *t*-tests when the sample size was small (e.g. *n* = 3 to six per group). Additionally, we used the mixed effects models with random intercept to account for the repeated measures for each individual when 20 samples were used in the analysis. The outcome measure was the change in the biomarker and protein concentration. Pre-outcome measure, diet (MMKD versus AHAD), experimental group (CN versus MCI), and the interaction between diet and experimental group were included in the model. The least squares mean for changes in biomarkers was calculated for each diet and experimental group. Testing whether the least squares mean was equal to 0 was the same as testing whether adjusted pre- and post-biomarkers were equal. The comparison of MCT2 expression on sEV^L1CAM^between responders and non-responders was performed using the two-sample *t*-tests. Correlations between sEV^L1CAM^ markers and clinical parameters were calculated using Pearson correlation coefficient estimates. Further, we performed logistic regression analyses considering responder status (responders versus non-responders) as the outcome variable and sEV^L1CAM^ as the independent variable. The area under the curve was estimated to examine the prediction ability of sEV^L1CAM^ on responder status. The non-parametric method was used to evaluate whether the fitted model (including sEV^L1CAM^ as a covariate) was better than the uninformative model (no covariate, null model).^35^ The correlations among sEV^L1CAM^, sEV^SYP^ and sEV^NCAM^ for NfL and Aβ1–42 were computed using Pearson correlation coefficient estimates. Multiple comparisons were not corrected because the study is mainly for descriptive purposes.

### Data availability

All data generated or analysed during this study are included in this published article. Further detail of methodologies is available from the corresponding author upon reasonable request. Requests for materials should be addressed to GD.

## Results

### MMKD and AHAD did not affect the total extracellular vesicles in the plasma

**Figure 1:**
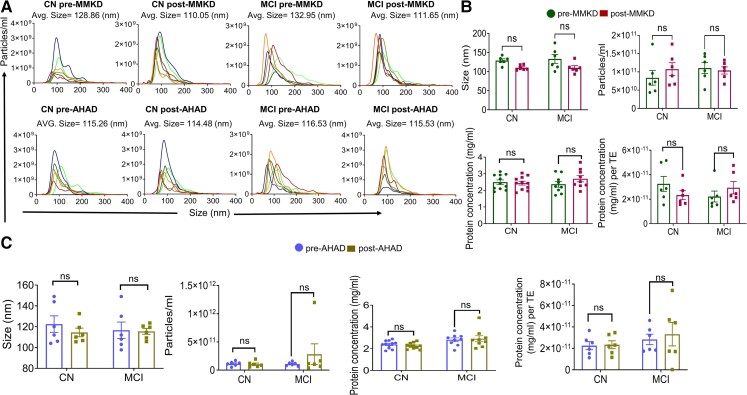
**Characterization of TE**. TEs were isolated from the plasma of CN and MCI participants in both pre- and post-MMKD and AHAD conditions. Six random TE samples/groups were analysed for their size distribution and concentration by NTA. **(A)** A representative line graph is presented for each group depicting concentration and size distribution for each of the six samples by a unique colour, and the average size of TE is mentioned on the top of the graph. **(B and C)** The bar diagrams present the size and concentration (particles/ml) of TE as mean ± SEM of *n* = 6 samples. Protein concentration in TE for CN (*n* = 11) and MCI (*n* = 9) participants, both pre- and post-MMKD and AHAD is presented as mean ± SEM. Protein concentration per TE is presented in lower right panels (*n* = 6 each). Paired *t*-test was applied for comparing size, concentration and protein concentration per TE in pre- and post-measures and the mixed effects model with random intercept was applied for comparing TE protein concentration in pre- and post-measures.

**Figure 2:**
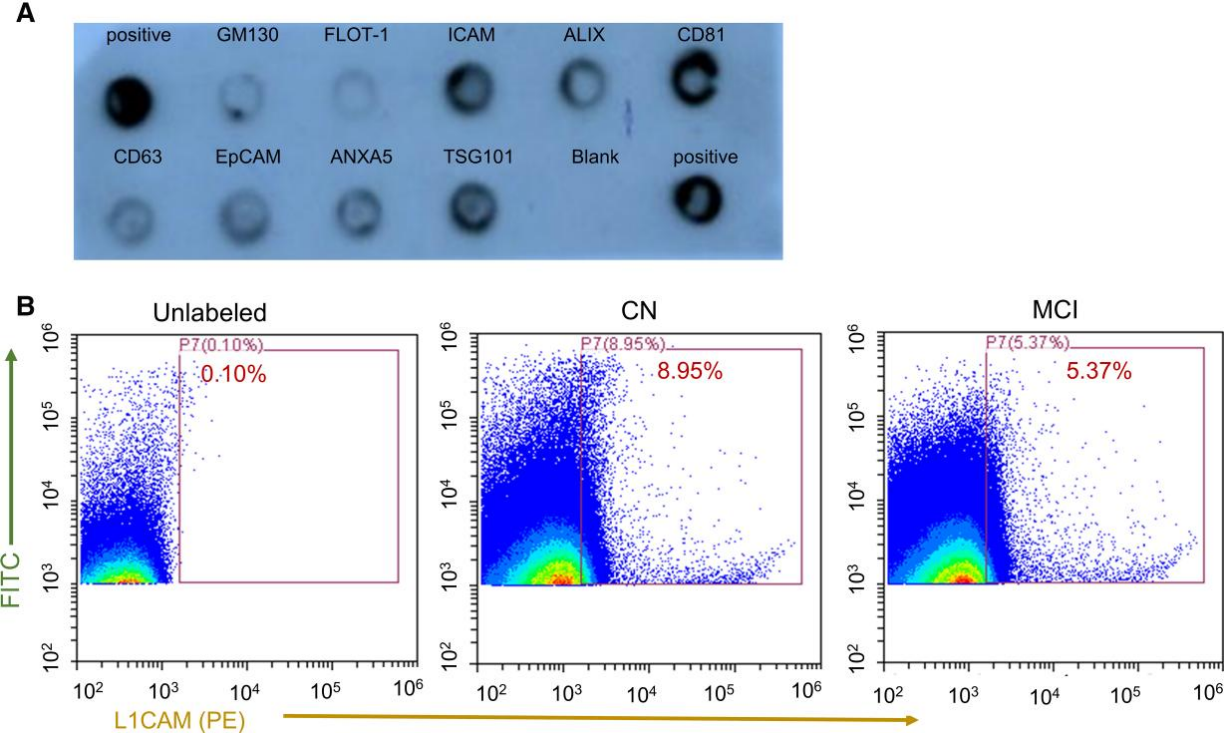
**Characterization of TE for EV and neuronal markers**. **(A)** TE were characterized by Exo-Check array (*n* = 3) for EV biomarkers. A representative blot is shown. **(B)** TE were analysed for surface L1CAM expression by flow cytometry. TE with only CellBrite 488 membrane dye (FITC) but without any other fluorescent antibody (unlabelled) were used as control (left panel). Twelve TE samples from CN and MCI groups were randomly selected and labelled with PE-tagged L1CAM antibody. TE were diluted with 1:100 folds in 0.22 µn filtered PBS and acquired on Cytoflex for 60 s. TE in the gated regions represents L1CAM + vesicles. Representative flow panels are shown for CN (middle panel) and MCI (right panel).

The nanoparticle tracking analyses (NTA) analysis confirmed that the isolated EV are in the size range of sEV with an average size of less than 150 nm. NTA analyses and protein quantification showed that neither MMKD nor AHAD significantly affected the size, concentration (particles/ml), protein loading and protein concentration per particle in TE (Fig. 1A–C). Array analysis showed the presence of exosomal biomarker proteins ICAM, ALIX, CD81, CD63, EpCAM, ANXA5 and TSG101 (Fig. 2A). Further, we analysed the percentage of sEV^L1CAM^ in TE and showed that sEV^L1CAM^ constitutes about 5–10% of TE (Fig. 2B). The key flow criteria and relevant controls, including the isotype and negative controls, for flow cytometry analyses of TE are described in Supplementary Fig. 2.

## The effect of MMKD and AHAD on sEV^L1CAM^ in the plasma

**Figure 3:**
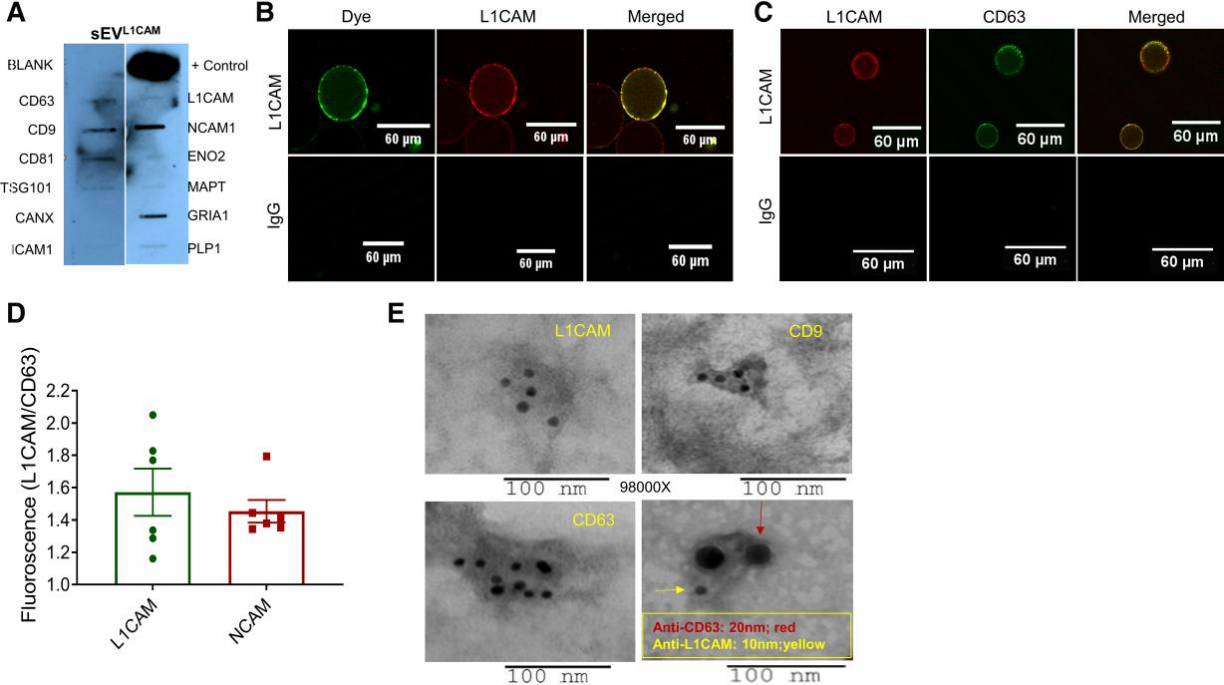
**Characterization of sEV^L1CAM^**. (**A)** sEV^L1CAM^ were characterized by Exo-Check (Neuro) array, and a representative blot is shown. **(B)** Confocal microscopy images of sEV^L1CAM^ attached on agarose resin and labelled with membrane labelling dye CellBrite and L1CAM fluorescent antibody (upper panel). Agarose resin tagged with IgG-biotin antibody, incubated with TE, and imaged with L1CAM-PE antibody and membrane labelling dye (lower panel) served as control. Scale bar is 60 μm. **(C)** sEV^L1CAM^attached to agarose resin were labelled with L1CAM-PE and CD63-AF647 fluorescent antibodies (upper panel). Agarose resin attached with IgG-biotin antibody were labelled using similar conditions as above (lower panel). Scale bar is 60 μm. **(D)** sEV^L1CAM^and sEV^NCAM^ were isolated using agarose beads tagged with L1CAM-biotin (*n* = 6) or NCAM-biotin (*n* = 6) antibodies, respectively. Intact sEV^L1CAM^ or sEV^NCAM^ (*n* = 6 each) (without lysis) were immobilized on CD63 antibody-coated ELISA plate in two sets. Next, one set was labelled with L1CAM-biotin antibody, and the other was with CD63-biotin and probed with streptavidin solution. The captured fluorescence from L1CAM-biotin wells was normalized with fluorescence from CD63-biotin wells and plotted as mean ± SEM. **(E)** Surface expression of biomarkers on sEV^L1CAM^ was assessed using a specific primary antibody (L1CAM) and gold-labelled secondary antibodies on CN (*n* = 4) and MCI group (*n* = 4). Co-expression of L1CAM and CD63 on sEV^L1CAM^ was confirmed using different-sized gold particle (10 nm or 20 nm) labelled antibodies. Red and yellow arrows represent CD63 and L1CAM, respectively. Representative TEM images are shown at 98 000×, and a scale bar is presented below each image.

We first characterized the purity, exosomal and neuronal characteristics of sEV^L1CAM^. Array analysis showed the expression of several neuronal biomarkers (L1CAM, NCAM1, ENO2, glutamate receptor ionotropic AMPA type subunit 1 (GRIA1) and PLP1) and established exosomal biomarkers (CD63, CD9, CD81 and TSG101) in sEV^L1CAM^ (Fig. 3A). sEV^L1CAM^ lacked CANX (calnexin), an ER protein usually absent in sEV/exosomes (Fig. 3A). Full blot for this array is presented in Supplementary Fig. 1B.

**Figure 4:**
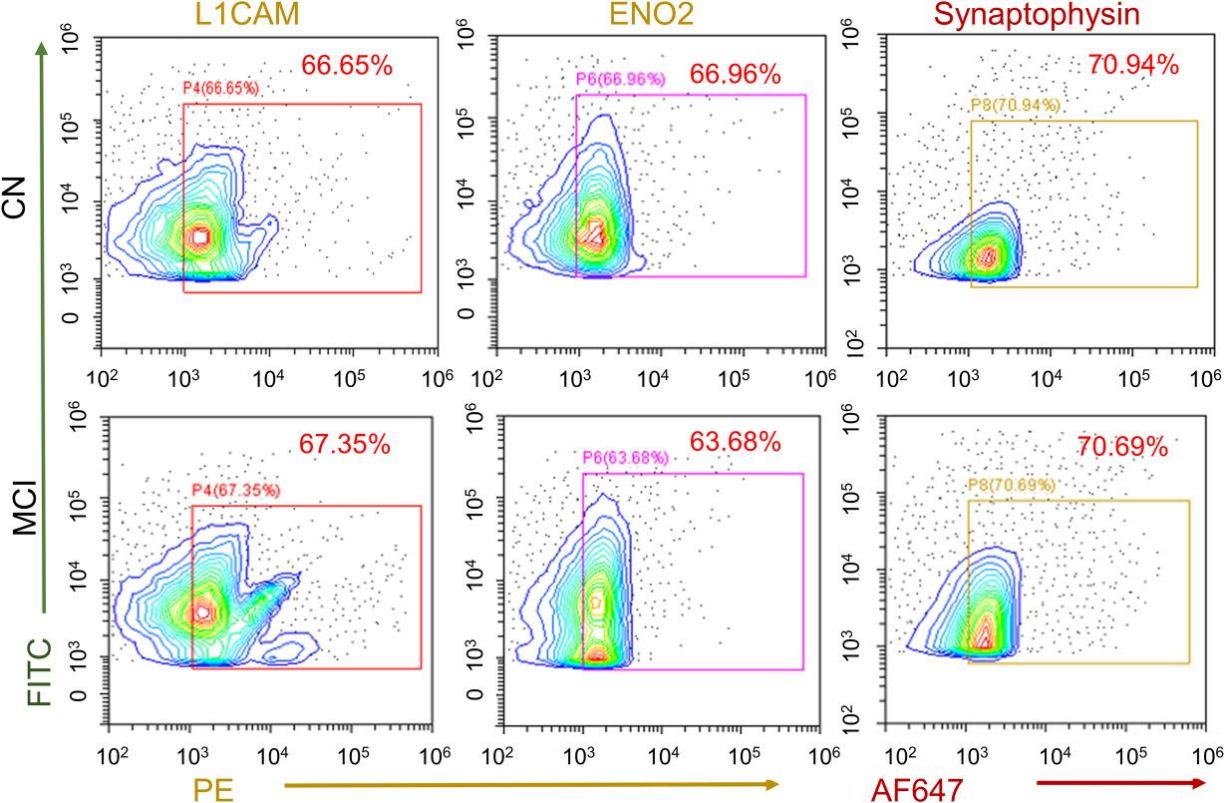
**Validation of purity of sEV^L1CAM^ by flow cytometry**. sEV^L1CAM^ from CN (*n* = 5) and MCI (*n* = 5) were characterized by flow cytometry. Right shift in the fluorescence in the gated region represents the L1CAM, ENO2 (PE) and synaptophysin (AF647) positive sEV^L1CAM^.

To confirm that the L1CAM mediated isolation of particles are indeed sEV and not free L1CAM proteins, we stained sEV^L1CAM^ bound to agarose resin with membrane labelling dye and L1CAM-PE antibody. The co-expression (yellow colour) of membrane labelling dye (green) and L1CAM (pseudo-red colour) confirmed that the isolated particles are sEV^L1CAM^ as agarose resin tagged with IgG-biotin antibody and incubated with TE showed no signals (Fig. 3B). Moreover, the L1CAM and CD63 co-expression (yellow colour) was also confirmed following the pull-down of sEV^L1CAM^ on agarose resin and stained with fluorescent-tagged L1CAM (green colour) and CD63 (red colour) antibodies (Fig. 3C). No detectable fluorescent signal in control beads (with IgG antibody) was observed, which confirmed the specific signals of L1CAM and CD63 on sEV^L1CAM^. Further, co-expression of L1CAM with CD63 on sEV^L1CAM^ was analysed by ELISA assay on CD63-antibody-coated ELISA plate (Fig. 3D). Higher normalized fluorescence with L1CAM-biotin antibody with sEV^L1CAM^and sEV^NCAM^showed the purity of isolation and that L1CAM co-expressed with NCAM and CD63. Immunogold labelling and TEM analyses further confirmed the presence of L1CAM on the surface of the sEV^L1CAM^ (Fig. 3E, upper left panel). sEV^L1CAM^ were also analysed for exosomal biomarkers CD9 and CD63. Size and co-expression of L1CAM and CD63 on sEV^L1CAM^ surface further confirmed that these vesicles are sEV (Fig. 3E, lower right panel). Next, TE and sEV^L1CAM^ were characterized by flow cytometry for L1CAM surface expression as well as 2 neuronal biomarkers (ENO2 and synaptophysin). TE analysis showed that similar to sEV^L1CAM^, sEV^SYP^also constitute about 5–10% of TE (Supplementary Fig. 2F). Moreover, isolated sEV^L1CAM^ showed >65% positivity for L1CAM, ENO2 and synaptophysin (Fig. 4). The key flow criteria and relevant controls, including the isotype control, for flow cytometry analyses of TE and NEE are described in Supplementary Figs 2 and 3.

**Figure 5:**
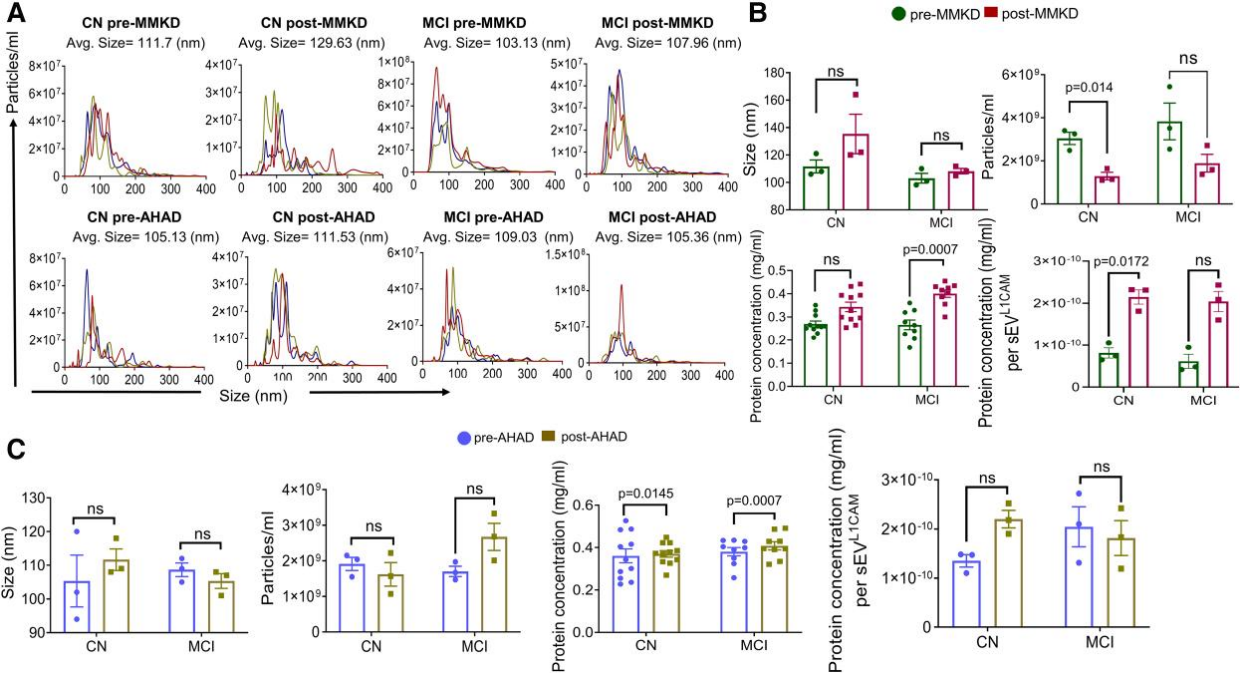
**The effect of MMKD and AHAD on the size, concentration and protein concentration of sEV^L1CAM^**. **(A)** sEV^L1CAM^ isolated from the TE of CN and MCI participants, both pre- and post-MMKD and AHAD (*n* = 3 each), were analysed for their size distribution and concentration by NTA. A representative line graph is shown for each group depicting concentration and size distribution for each of the three samples by a unique colour, and the average size of sEV^L1CAM^ is mentioned on the top of the graph. The bar diagrams **(B and C)** present the size and concentration of sEV^L1CAM^ as mean ± SEM of *n* = 3 samples. Protein concentration in sEV^L1CAM^ for CN (*n* = 11) and MCI (*n* = 9) participants, **(B)** both pre- and post-MMKD and **(C)** AHAD is presented as mean ± SEM. Protein concentration per sEV^L1CAM^ is presented in the right panels (*n* = 3 each). Paired *t*-test was applied to compare sEV^L1CAM^size, concentration and protein concentration per sEV^L1CAM^ in pre- and post-measures. The mixed effects model with random intercept was applied for comparing sEV^L1CAM^ protein concentration in pre- and post-measures.

Next, NTA analysis was performed to analyse the average concentration and mean size of sEV^L1CAM^. NTA confirmed the mean size of sEV^L1CAM^between 100 and 150 nm which represents the size range of sEV (Fig. 5A). Further, neither MMKD nor AHAD significantly affected the size of sEV^L1CAM^ (Fig. 5B and C). sEV^L1CAM^ concentration (particles/ml) decreased significantly in CN with MMKD, but no statistically significant change was observed in MCI or with AHAD (Fig. 5B and C). Interestingly, total protein concentration significantly increased in MCI following MMKD intervention while protein concentration per sEV^L1CAM^ was significantly increased in CN group. Total protein concentration increased following AHAD in both CN and MCI groups; however, no change in protein concentration per sEV^L1CAM^ was noted after AHAD (Fig. 5B and C).

## The effect of MMKD and AHAD on ADRD biomarkers in sEV^L1CAM^

**Figure 6:**
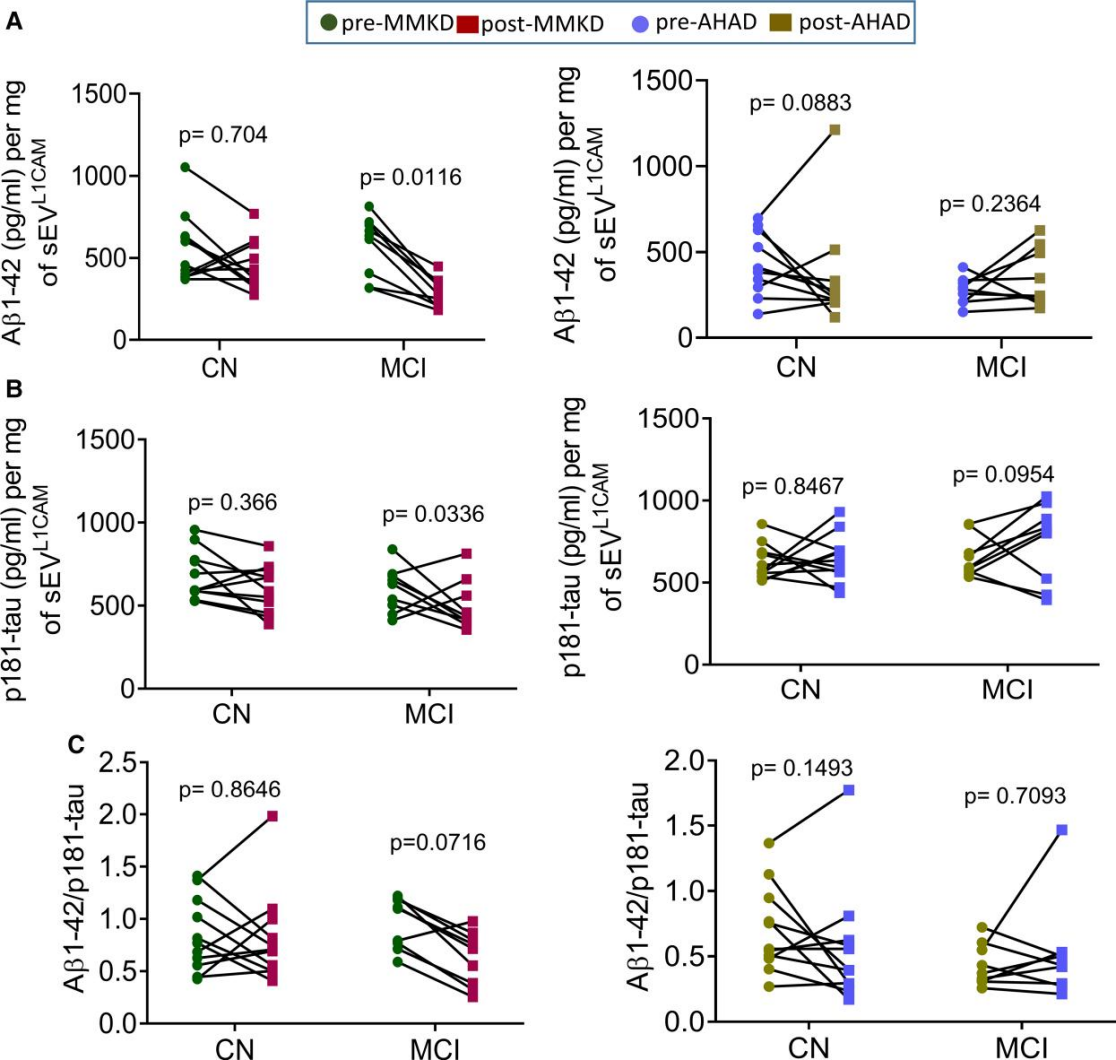
**The effect of MMKD and AHAD on Aβ1–42 and p181-tau**. sEV^L1CAM^ from CN (*n* = 11) and MCI (*n* = 9) participants both pre- and post-MMKD and AHAD were analysed for various ADRD biomarkers by ELISA for **(A)** Aβ1–42, **(B)** p181-tau and **(C)** Aβ1–42/p181-tau. Each biomarker concentration (pg/ml) was presented as per mg sEV^L1CAM^ protein concentration (mean ± SEM). The mixed effects model with random intercept was applied for comparing pre- and post-measures.

**Figure 7:**
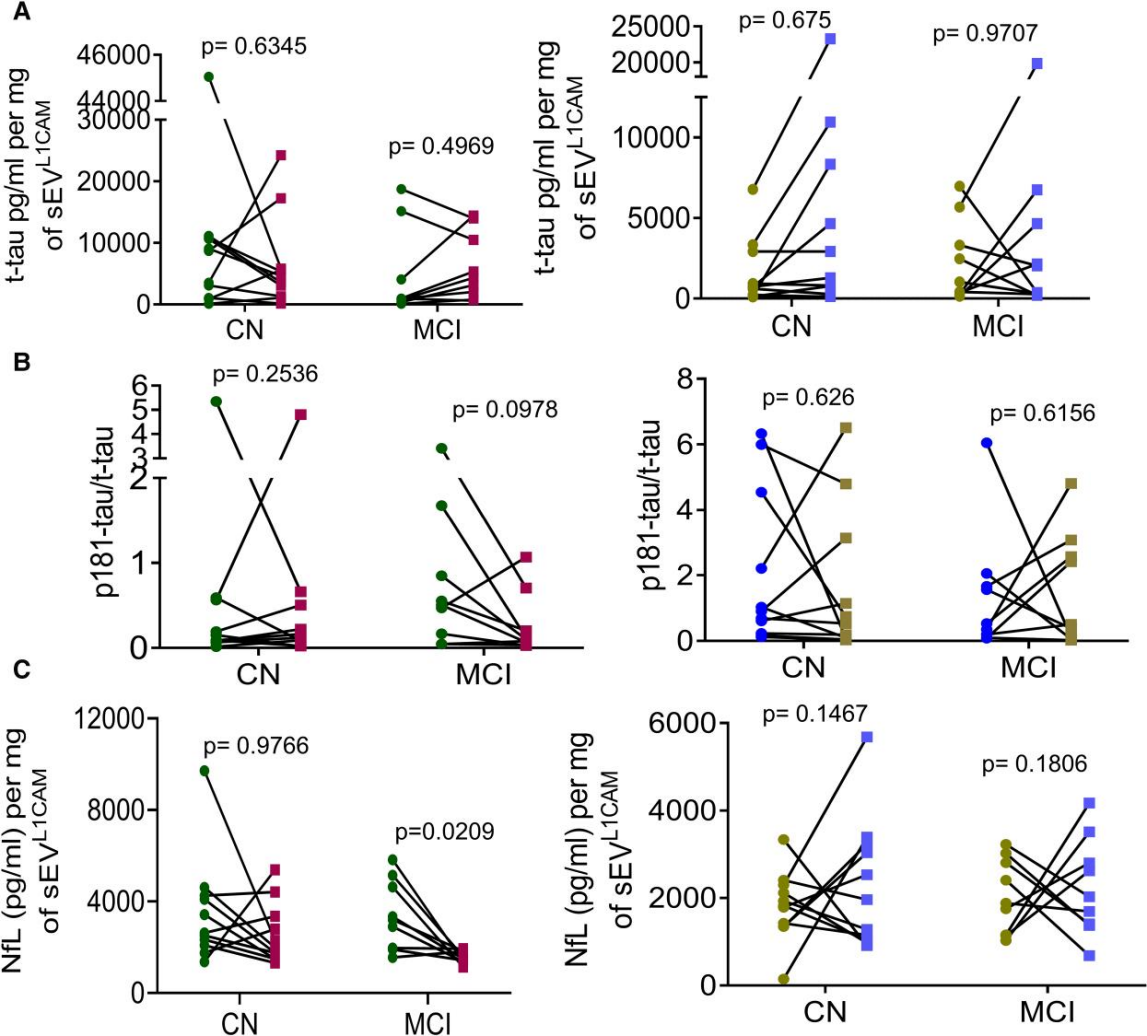
**The effect of MMKD and AHAD on tau and NfL**. sEV^L1CAM^ from CN (*n* = 11) and MCI (*n* = 9) participants both pre- and post-MMKD and AHAD were further analysed for **(A)** t-tau, **(B)** p181-tau/t-tau and **(C)** NfL. Each biomarker concentration (pg/ml) was presented as per mg sEV^L1CAM^ protein concentration (mean ± SEM). The mixed effects model with random intercept was applied for comparing pre- and post-measures.

Next, we characterized the sEV^L1CAM^ for various ADRD biomarkers (Figs 6 and 7). The MMKD intervention significantly reduced the Aβ1–42 level in the MCI group while no significant effect was observed with AHAD (Fig. 6A). Similarly, MMKD showed a reduced trend of p181-tau in CN (8/11) and significant reduction in MCI (6/9) groups with a mean reduction of 34.9% (*P* = 0.033) (Fig. 6B). A decrease trend in Aβ1–42/p-181tau ratio (8/9) was observed following MMKD treatment in MCI participants (Fig. 6C). No significant effect of MMKD or AHAD was observed on t-tau (Fig. 7A). However, a trend towards reduction was observed in the p-181tau/t-tau ratio in MCI (7/9) groups (though statistically not significant) with an average decrease of 80% observed following MMKD intervention; no such effect was observed with AHAD (Fig. 7B). Lastly, MMKD significantly reduced the NfL level in sEV^L1CAM^ from the MCI group, while no significant effect was observed with the AHAD (Fig. 7C).

## The effect of MMKD and AHAD on glutamate-glutamate receptors in sEV^L1CAM^

**Figure 8:**
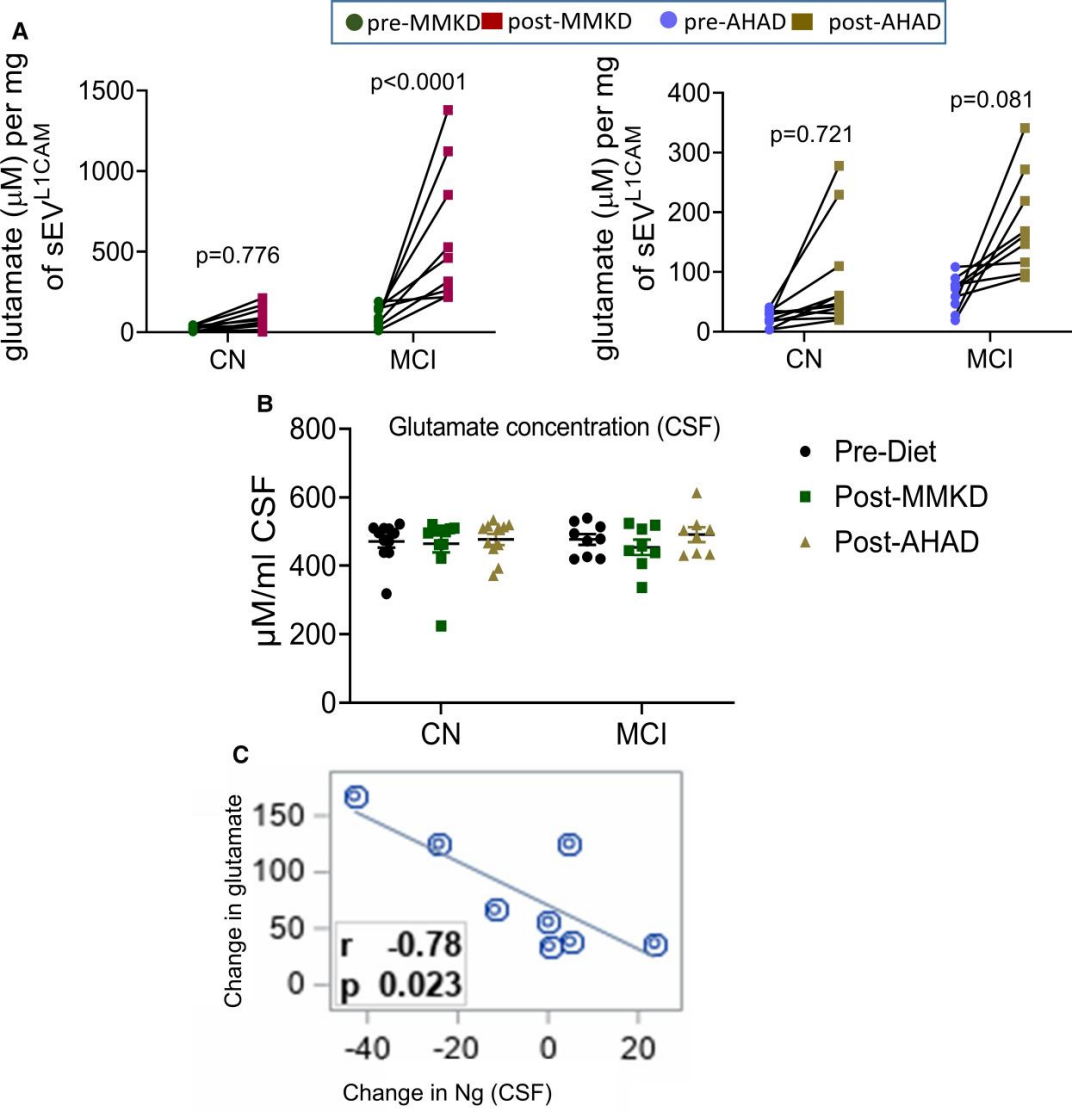
**The effect of MMKD and AHAD on glutamate**. sEV^L1CAM^ from CN (*n* = 11) and MCI (*n* = 9) participants both pre- and post-MMKD and AHAD, were analysed for glutamate and glutamate receptors. **(A)** sEV^L1CAM^ were lysed and analysed for glutamate levels. Total glutamate concentration was normalized with sEV^L1CAM^ protein concentration and represented as µM glutamate per mg of sEV^L1CAM^ (mean ± SEM). The mixed effects model with random intercept was applied for comparing pre- and post-measures. **(B)** Glutamate concentration in CSF samples presented as µM/ml of CSF (mean ± SEM). Repeated measure analysis of variance was used to calculate the statistical difference in CN and MCI groups. For CN, *F* = 0.10, d.f. = (2,30), *P* = 0.91 and for MCI, *F* = 0.86, d.f. = (2,22), *P* = 0.44). **(C)** Correlation of sEV^L1CAM^ glutamate with CSF neurogranin.

Next, we characterized sEV^L1CAM^ for glutamate levels and expression of various glutamate receptors (GRIN1, GRIN2A, glutamate receptor ionotropic NMDA2B (GRIN2B) and GRIA1). The glutamate level in sEV^L1CAM^ was increased after MMKD among both CN and MCI groups (with a statistically significant increase in MCI group); a similar but less prominent pattern (statistically non-significant) was found in both CN and MCI groups with AHAD (Fig. 8A). Interestingly, no change in the glutamate levels in CSF was observed (Fig. 8B). Importantly, changes in glutamate expression in sEV^L1CAM^ in the CN group from pre- to post-MMKD negatively correlated with corresponding changes in Ng concentration in CSF^17^ (*n* = 8) (Fig. 8C), an established biomarker of synaptic plasticity and long-term potentiation.^36,37^ A similar negative correlation between changes in sEV^L1CAM^-glutamate and CSF-Ng was also observed for the MCI group (post-MMKD) but did not achieve statistical significance, likely due to the low number of samples in which Ng could be reliably measured (r = −0.65, *P* = 0.55) (data not shown).

**Figure 9:**
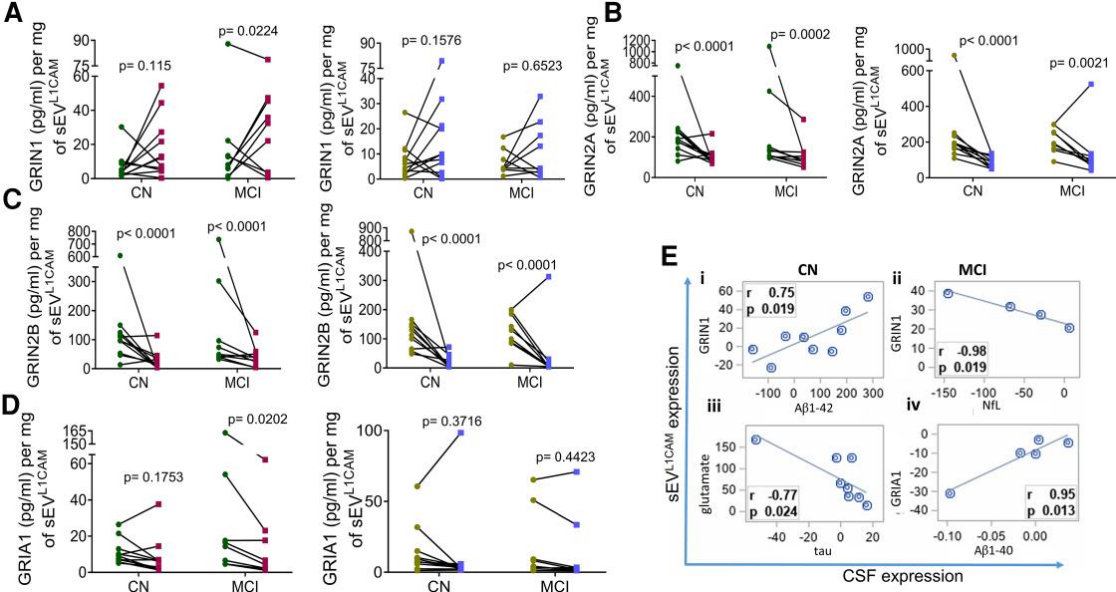
**The effect of MMKD and AHAD on glutamate receptors**. sEV^L1CAM^ from CN (*n* = 11) and MCI (*n* = 9) participants both pre- and post-MMKD and AHAD, were analysed for various glutamate receptors. **(A–D)** sEV^L1CAM^ were lysed with RIPA buffer and analysed for the concentration of glutamate receptors (GRIN1, GRIN2A, GRIN2B and GRIA1) by ELISA and presented as pg/ml per mg of sEV^L1CAM^ (mean ± SEM). The mixed effects model with random intercept was applied for comparing pre- and post-measures. **(E)** Pearson correlation coefficients between changes in sEV^L1CAM^ (MMKD) from CN and MCI with corresponding changes in CSF measures are presented.

Interestingly, MMKD showed a significant increased expression of GRIN1 (or GluN1) in MCI groups and an increased trend in CN (7/11); a similar but less prominent (statistically non-significant) trend in GRIN1 expression was also observed in the AHAD group (Fig. 9A). Compared with GRIN1, a significant decrease was observed in the expression of GRIN2A (Fig. 9B) and GRIN2B with both MMKD and AHAD (Fig. 9C). Though, a significant decrease in GRIA1 was observed only with MMKD in MCI group (Fig. 9D).

Importantly, in sEV^L1CAM^ (MMKD) from the CN group, diet-induced changes in GRIN1 showed strong positive correlation with CSF Aβ1–42 changes^17^ (*r* = 0.75; *P* = 0.019), and glutamate showed strong negative correlation with CSF tau^17^ (*r* = −0.77; *P* = 0.024) (Fig. 9E). Similarly, in sEV^L1CAM^ (MMKD) from MCI group, GRIN1 changes showed strong negative correlations with CSF NfL changes^17^ (*r* = −0.98; *P* = 0.019), an established biomarker for neurodegeneration; and GRIA1 showed strong positive correlation with Aβ1-40^17^ (*r* = 0.95; *P* = 0.013) (Fig. 9E). Furthermore, change in GRIN1 expression in sEV^L1CAM^ (MMKD) negatively correlated with change in CSF Ng for MCI, but this correlation did not reach statistical significance (r = −0.62 *P* = 0.57) (data not shown). Finally, the change in expression of different glutamate receptors (GRIN1, GRIA1, GRIN2A, GRIN2B) was correlated with change in the levels of analysed Alzheimer’s disease-related biomarkers in sEV^L1CAM^ and outcomes are present in Supplementary Tables 2 and 3.

## The effect of MMKD and AHAD on AGEs and inflammation

**Figure 10:**
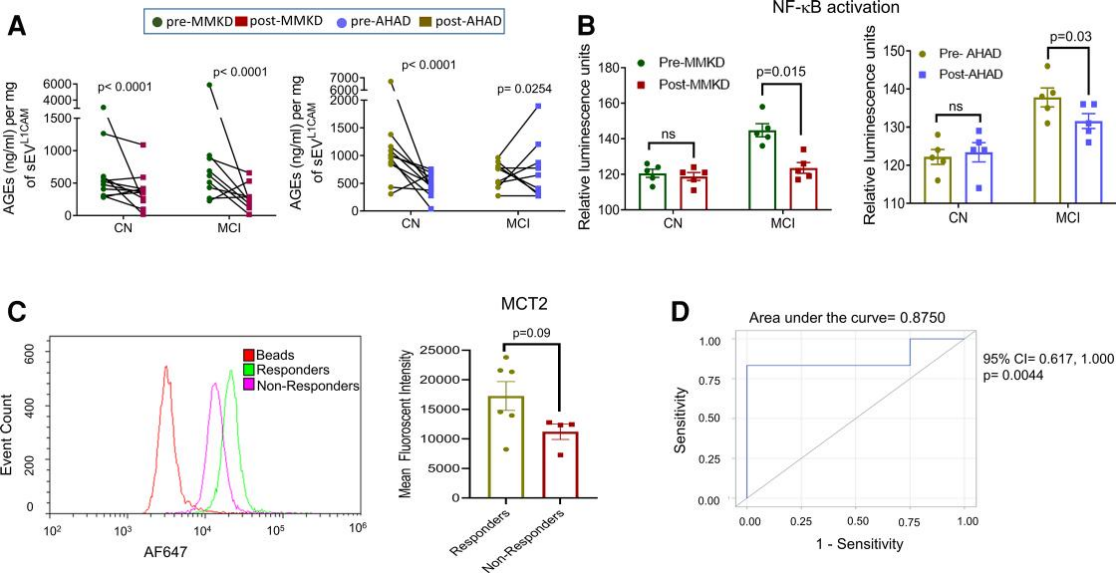
**The effect of MMKD on inflammatory response and predictability of MCT2 expression on sEV^L1CAM^for MMKD effect**. sEV^L1CAM^ from CN (*n* = 11) and MCI (*n* = 9) participants both pre- and post-MMKD and AHAD diet were analysed for the following: **(A)** The concentration of AGEs was analysed by ELISA assay, and the final concentration was calculated from the standards and normalized with sEV^L1CAM^ protein concentration. The final concentration of AGEs was represented as ng/ml per mg sEV^L1CAM^ (mean ± SEM). The mixed effects model with random intercept was applied for comparing pre- and post-measures. **(B)** NF-κB activation assay in THP-1 lucia monocyte cells was performed as described in the methods. sEV^L1CAM^ from both CN and MCI participants in both pre- and post-MMKD and -AHAD conditions were pooled randomly to make five replicates/group. THP-1 lucia cells were incubated with sEV^L1CAM^ and analysed for NF-κB activation. Graphs were plotted from five independent replicates (mean ± SEM). Paired *t*-test was applied to compare pre- and post-measures. **(C)** The expression of MCT2 was analysed on sEV^L1CAM^ surface by flow cytometry. sEV^L1CAM^ were isolated using biotin-labelled L1CAM antibody tagged on streptavidin-coated magnetic beads and further labelled with MCT2 AF647 antibody. Magnetic beads were analysed by flow by acquiring 10 000 events per sample. Mean fluorescent intensities (MFI) were analysed by FCS Express software. **(D)** Area under the curve of MCT2 sEV^L1CAM^ and responders/non-responders for Aβ1–42 reduction was plotted. A two-sample *t*-test was applied to compare responders versus non-responders.

Statistically significant decreased levels of AGEs following MMKD was observed in both CN and MCI groups (Fig. 10A, left panel), a similar but less prominent trend was also observed with AHAD (Fig. 10A, right panel). Next, the effect of sEV^L1CAM^ from CN and MCI groups (pre- and post-MMKD and AHAD) was assessed on NF-κB activity in human THP-1 monocytes as a molecular surrogate for inflammation as we recently reported.^33^ sEV^L1CAM^ from the MCI group showed a higher baseline level of NF-κB activity than the CN group prior to starting interventions. A decrease in the activity of secreted luciferase, suggesting a strong reduction of NF-κB activation, was observed in monocytes treated with sEV^L1CAM^ from MCI participants after the MMKD intervention, suggesting an anti-inflammatory effect of MMKD (Fig. 10B, left panel). A similar pattern was also seen with sEV^L1CAM^ from MCI participants after the AHAD intervention (Fig. 10B, right panel).

### MCT expression on sEV^L1CAM^ could predict response to MMKD

Next, we characterized MCT1 and 2 expressions on sEV^L1CAM^ surface by flow cytometry. Interestingly, MCT2 expression was higher than MCT1 (data not shown), which is supported by previous reports showing higher expression of MCT2 in neurons.^38,39^ Importantly, the participants that showed Aβ1–42 decrease (an average decrease of 45%) in sEV^L1CAM^ following MMKD (responders) exhibited a trend for higher MCT2 expression on sEV^L1CAM^ prior to the MMKD intervention (Fig. 1C). Moreover, the expression of MCT2 on sEV^L1CAM^ prior to MMKD clearly differentiated ‘responders’ and ‘non-responders’ to predict the impact of MMKD on Aβ1–42 expression with the area under the curve of 0.8750 (*P* = 0.0044, 95% confidence interval 0.617, 1.000) (Fig. 10D).

## The effect of MMKD on ADRD biomarkers is consistent irrespective of the choice of surface markers used to isolate NEE

**Figure 11:**
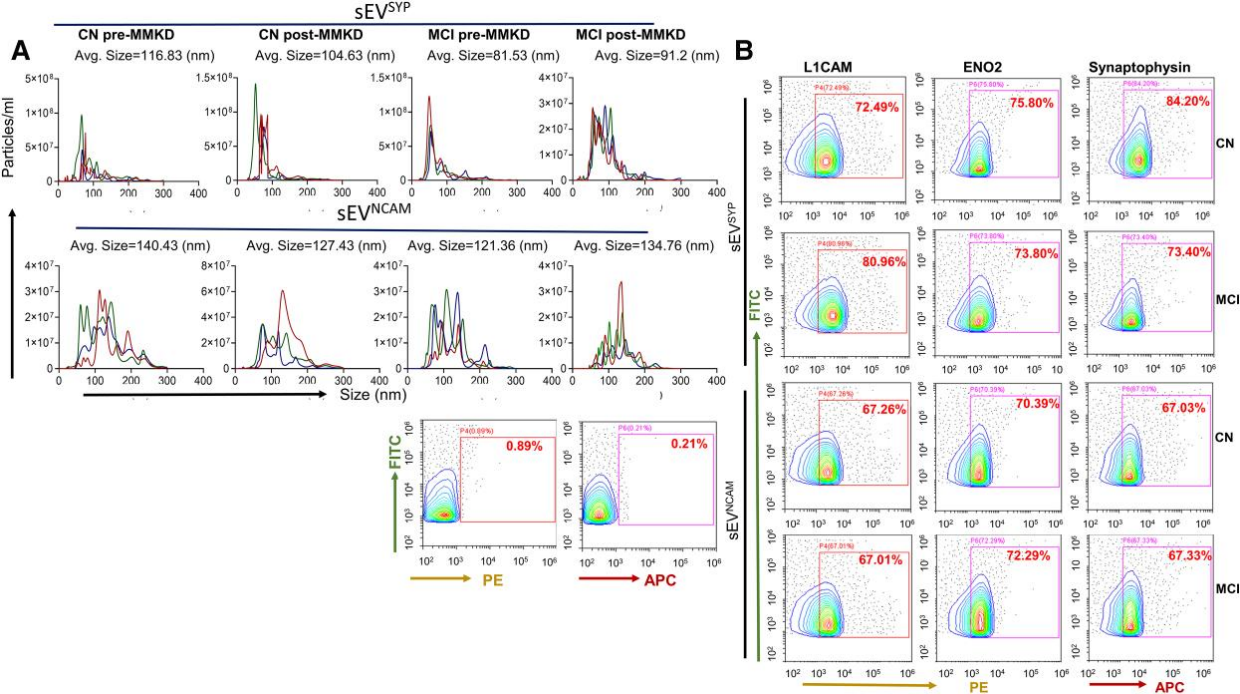
**Characterization of NEE isolated using different surface biomarkers**. NEE (sEV^SYP^and sEV^NCAM^) were isolated from TE from both CN (*n* = 5) and MCI (*n* = 5) participants in pre- and post-MMKD conditions using biotin-labelled synaptophysin or NCAM antibodies. **(A)** A representative line graph is presented for each group (Top panel: sEV^SYP^; bottom panel: sEV^NCAM^) depicting concentration and size distribution for each of the three samples by a unique colour and the average size of NEE is mentioned on the top of the graph. **(B)** sEV^SYP^and sEV^NCAM^ were analysed for L1CAM, ENO2 and synaptophysin by flow cytometry.

**Figure 12:**
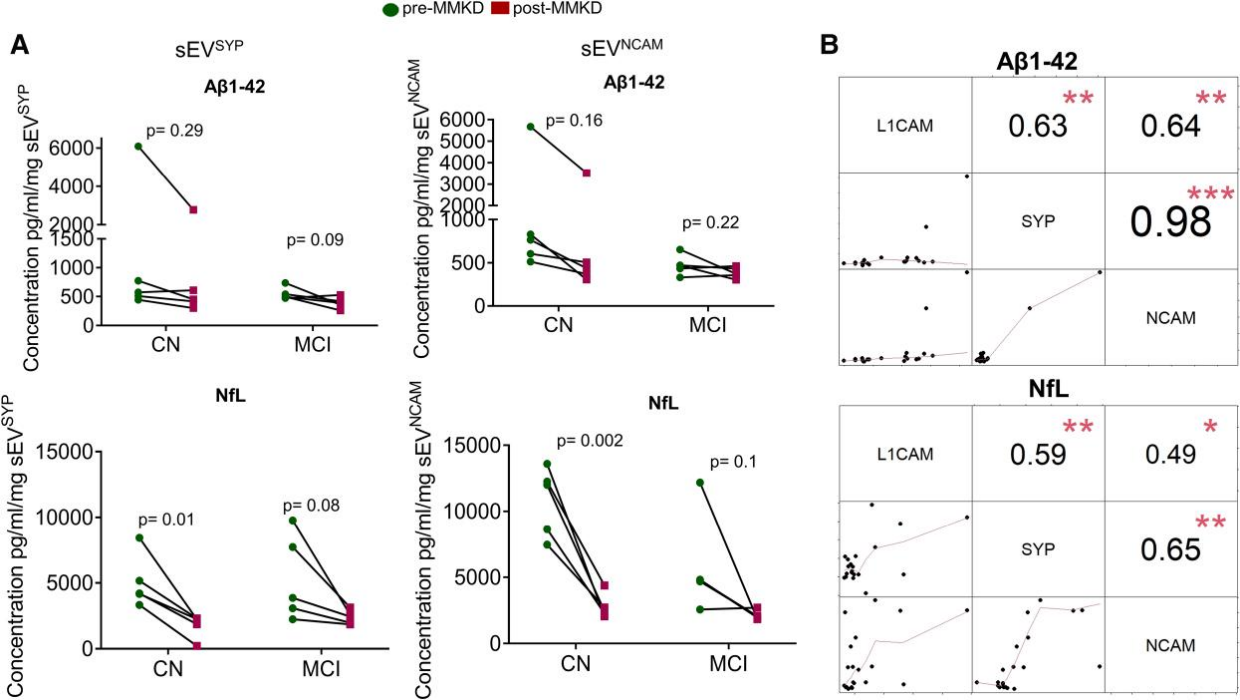
**Validation of results in plasma NEE. (A)** sEV^SYP^and sEV^NCAM^ were analysed for Aβ1–42 and NfL in both pre- and post-MMKD by ELISA. Final concentrations are presented as pg/ml per mg of sEV^SYP^or sEV^NCAM^. Paired *t*-test was applied to compare pre- and post-measures. **(B)** The matrix of correlation plots with smoothed regression lines for NfL and Aβ1–42. Pearson correlations with the corresponding significance levels are presented (*P < 0.05, **P < 0.01, ***P < 0.001).

Lastly, we isolated NEE (sEV^SYP^ and sEV^NCAM^) from plasma using two additional surface markers, synaptophysin and NCAM; and assessed the impact of MMKD on the levels of Aβ1–42 and NfL (Figs 11 and 12). NTA characterization revealed that the size distribution and concentration of sEV^SYP^ and sEV^NCAM^ were not significantly altered following MMKD intervention (Fig. 11A and Supplementary Fig. 4). Flow cytometry analysis on isolated EV also confirmed the enrichment and purity of NEE (sEV^SYP^ and sEV^NCAM^) (Fig. 11B).

A decreased trend was observed with MMKD intervention in sEV^SYP^ (*n* = 5; CN: 4/5; MCI: 4/5) and sEV^NCAM^ (*n* = 5; CN: 5/5, MCI: 3/5) for Aβ1–42 level (Fig. 12A, upper panels). Similarly, NfL level was also decreased following MMKD intervention in sEV^SYP^ and sEV^NCAM^ for all samples (Fig. 12A, lower panels). The correlations among sEV^L1CAM^, sEV^SYP^ and sEV^NCAM^ for Aβ1–42 were all above 0.63 (all *P*-values <0.01). Especially the correlation between sEV^SYP^ and sEV^NCAM^ was high (0.98 with *P*-value <0.001) (Fig. 12B, upper panel). The correlations among sEV^L1CAM^, sEV^SYP^ and sEV^NCAM^ for NfL were all above 0.49 (all *P*-values <0.05) (Fig. 12B, lower panel). This shows that levels of NfL and Aβ1–42 in sEV^L1CAM^, sEV^SYP^ and sEV^NCAM^ are moderate to highly correlated.

## Discussion

Alzheimer’s disease is a fatal neurodegenerative disorder with only limited disease-modifying treatment or preventative intervention, and its incidences are expected to triple by 2050.^40,41^ To accelerate preventive approaches against Alzheimer’s disease, novel tools are needed which are non/less-invasive and may be used repetitively over a period of time, with a goal to assess treatment response and/or screen participants before the start of intervention for targeted therapy. EV in biofluids could serve such a purpose. Here, we characterized NEE to understand the molecular effects underlying the efficacy of MMKD in Alzheimer’s disease utilizing archived biofluids from the pilot clinical study,^17^ which previously showed a positive impact of MMKD intervention on CSF Alzheimer’s disease biomarkers. MMKD reduced the levels of neurodegeneration markers, increased glutamate and differentially altered the expression of glutamate receptors in NEE. Importantly, we evaluated the potential of MCT2 on sEV^L1CAM^ for identifying the participants exhibiting reduced Aβ1–42 levels following MMKD intervention. Since the expression of MCT2 is more on neurons and possesses a higher affinity towards substrate compared with MCT1,^14^ higher expression of MCT2 on neurons may be expected to increase transport of ketone bodies and help neurons to make a metabolic transition for the substrate. Thus, assessing the expression of MCT2 on plasma NEE may be an important measure in determining the effectiveness of KD and help screen participants that may benefit from such an intervention.

Neuronal EV have shown promise in detecting established Alzheimer’s disease biomarkers such as Aβ1–42.^28^ Higher Aβ1–42 levels in plasma neuronal EV have been shown to correspond to greater amyloid burden assessed with PET (PiB tracer), and Aβ1–42 levels in neuronal EV directly correlate with Alzheimer’s disease stage and progression.^21,28,42^ This is in contrast to the well-established finding that Aβ1–42 levels in the CSF are inversely correlated with neuritic plaque burden.^43^ Furthermore, the findings regarding Alzheimer’s disease biomarkers in MCI have been mixed. CSF Aβ and p-tau levels have been shown to be comparable in CN and MCI in several studies.^44,45^ We previously reported that MMKD treatment increased the Aβ1–42 levels in the CSF of participants, suggesting a reduction in Aβ aggregation and better clearance.^17^ Here, we observed a significant reduction in sEV^L1CAM^ Aβ1–42 levels in those same MCI participants following MMKD treatment. This is an exciting observation suggesting the sEV^L1CAM^ in plasma could be useful in characterizing Aβ plaques in the brain and could be an additional blood-based parameter to potentially supplement the existing neuroimaging and CSF measures. However, this certainly warrants the need for a further study into the mechanism involved in the loading of Aβ in EV and their secretion. It is plausible that Aβ in EV is mainly the intracellular Aβ that is known to arise from APP processing in the endosomal compartment.^46^ This speculation is supported by the fact that neuronal EV cargo includes BACE1,^23^ which plays a critical role in APP processing and the generation of Aβ, both at the cell membrane as well in endosomes.^46^ sEV^L1CAM^ also showed a significant reduction in p181-tau levels following MMKD intervention, as well as a reduction in p181-tau/total tau from most participants (7/9) in the MCI group. Also notable was the observation that sEV^L1CAM^ had significantly decreased NfL levels in the MCI group following MMKD intervention, even though prior CSF data was unrevealing.^17^ These results suggest that sEV^L1CAM^ in plasma could provide valuable information about the changes in the key Alzheimer’s disease biomarkers.

Alterations in cerebral glucose and glutamate levels can lead to the deposition of Aβ plaques.^47^ KD may compensate for glucose hypometabolism and restore mitochondrial bioenergetics; however, its effect on neuronal glutamate levels remains unknown. Importantly, over 40% of neuronal synapses are glutamatergic, and the disturbance in the glutamate levels and glutamate receptors expression and their localization have been implicated in the pathophysiology of Alzheimer’s disease, influencing memory, cognition and behaviour.^47^ We observed a significant increase in the glutamate level in sEV^L1CAM^ following MMKD intervention in MCI participants, suggesting higher intracellular glutamate in the neurons. However, no change in the CSF glutamate level was observed, highlighting the importance of studying NEE. An earlier study has reported that plasma exosomes reflect a similar change in the expression of glutamate receptor with aging as in the brain tissue of transgenic mice.^48^ Further, MMKD targeted the expression of various ionotropic glutamate receptors, consisting of NMDA and AMPA type, suggesting a restoration of glutamate-glutamate receptor signalling, which is critical for long-term potentiation. For example, we observed an increased expression of GRIN1, an obligatory subunit of NMDA type glutamate receptor, while observing a decrease in the GRIN2A and GRIN2B subunits of NMDA receptors and a decrease in AMPA subunit GRIA1. It is not clear, though, whether MMKD intervention has an inhibitory effect on the expression of these glutamate receptors subunits in the extrasynaptic region, which is usually associated with higher Aβ production and neuropathology of Alzheimer’s disease.^49^ Aβ also adversely affects glutamate cycling and glutamate receptor signalling while reducing the uptake of glutamate by astrocytes and promoting higher glutamate levels in the synaptic cleft and activation of extrasynaptic GRIN2B containing NMDA receptors leading to synaptic impairment.^47^ Thus, a ketogenic intervention may target Aβ expression at multiple levels and possibly its reduction coincides with the restoration of glutamatergic neurons.

AGEs are formed by non-enzymatic glycosylation of macromolecules and increase in response to hyperglycaemia and oxidative stress.^50^ Higher AGEs could induce toxicity via aberrant cross-linking with proteins and the production of ROS.^51,52^ AGEs are pro-inflammatory, activate RAGE (receptor for AGE), contribute to Aβ production in the brain, and regulate the influx of circulating Aβ across the blood–brain barrier.^53,54^ AGE content in neurons (and astrocytes) increases with increasing Braak tangle stage, CERAD tangle score and neuritic plaques.^50^ RAGE also promotes senile plaque formation via tau hyperphosphorylation, synaptic dysfunction and neuronal death.^53,55–57^ We observed a substantial reduction in AGEs in sEV^L1CAM^ following MMKD intervention that correlated well with the significant reduction observed in the sEV^L1CAM^ induced monocyte NF-κB activity.

Although L1CAM has been extensively used to isolate NEE and characterize neuronal biomarkers,^21,22,24,25,27,30^ several questions have been recently raised regarding its presence on EV surface,^32^ as well as the neuronal specificity of L1CAM. The present study addresses a few of these critical questions. We have presented immunogold labelling/TEM, confocal microscopy fluorescence images, modified ELISA-based assay and flow cytometry data to confirm the presence of L1CAM on the surface of sEV. We further isolated NEE from plasma-based upon two more surface biomarkers (synaptophysin and NCAM) and characterized Aβ1–42 and NfL levels. Using both the markers, we observed quite similar trend in terms of the effect of MMKD on the levels of Aβ1–42 and NfL as with L1CAM. Further, we also identified the relative percentage of sEV^L1CAM^and sEV^SYP^ in plasma. Overall, though high concordance was observed in results between sEV^L1CAM^, sEV^SYP^ and sEV^NCAM^ but outcomes were not exactly the same, suggesting a possible heterogeneity in the neuronal-derived EV populations, which needs to be further studied.

Despite several advancements, there are a few notable drawbacks of the present study. One of the major limitations is the small sample size and crossover design of the present study. This could be overcome by adopting a similar approach in other studies with a higher number of participants. Another inherent limitation of the present study could be that the use of above-mentioned neuronal surface biomarkers does not confirm 100% neuronal or CNS origin, though clearly showed enrichment for Alzheimer’s disease biomarkers. This warrants the need for more specific EV biomarkers for neurons and other cell types in the CNS. Besides, our assays did not provide information about the localization of cargo molecules whether present in the core, membrane or sticking on the surface of sEV. Lastly, sample storage and freeze/thaw conditions could potentially affect the samples and studied measures. However, prior to conducting this study, we have performed a pilot study to confirm that plasma storage (at −80°C) does not significantly affect the integrity and cargo of sEV^L1CAM^ compared with fresh samples.

Overall, results from the present study support the usefulness of plasma NEE as a tool in developing novel preventive and therapeutic interventions for Alzheimer’s disease and potentially related neurodegenerative disorders. The present study additionally sheds light on the beneficial effects of the MMKD on previously reported regulation of Alzheimer’s disease pathology^17^ and associated mechanistic pathways.

## Supplementary Material

fcac262_Supplementary_Data

## Data Availability

The data that support the findings of this study are available from the corresponding author, [GD], upon reasonable request.

## Abbreviations

AD =: Alzheimer’s disease
ADRD =: Alzheimer's disease and related dementias
AGE =: advanced glycation endproduct
AHAD =: American Heart Association diet
AUC =: area under the curve
Aβ =: amyloid-beta
CN =: cognitively normal
CSF =: cerebrospinal fluid
ENO2 =: Enolase 2
EV =: extracellular vesicles
KD =: ketogenic diet
L1CAM =: L1 cell adhesion molecule
LP =: lumbar puncture
MCI =: mild cognitive impairment
MCT =: monocarboxylate transporter
MMKD =: modified Mediterranean-ketogenic diet
MRI =: magnetic resonance imaging
NCAM =: neural cell adhesion molecule
NEE =: neuronal-enriched small extracellular vesicles
NfL =: neurofilament light
Ng =: neurogranin
NTA =: nanoparticle tracking analyses
PET =: positron emission tomography
RAGE =: receptor of AGE
sEV =: small extracellular vesicles
sEV^L1CAM^ =: L1CAM positive small EV
sEV^NCAM^ =: NCAM positive small EV
sEV^SYP^ =: synaptophysin positive small EV
SYP =: synaptophysin
TE =: total extracellular vesicles
TEM =: transmission electron microscopy
TMB =: 3,3′,5,5′-tetramethylbenzidine
VSSC =: violet side scatter
