# Supplementary material for: Small extracellular vesicles in plasma reveal molecular effects of modified Mediterranean-ketogenic diet in participants with mild cognitive impairment
Source: Brain Commun. 2022 Oct 19;4(6):fcac262. doi: 10.1093/braincomms/fcac262 (PMC9629368; doi:10.1093/braincomms/fcac262)
Supplement: fcac262_Supplementary_Data [file fcac262_supplementary_data.zip › Supplementary_Figures.pdf]

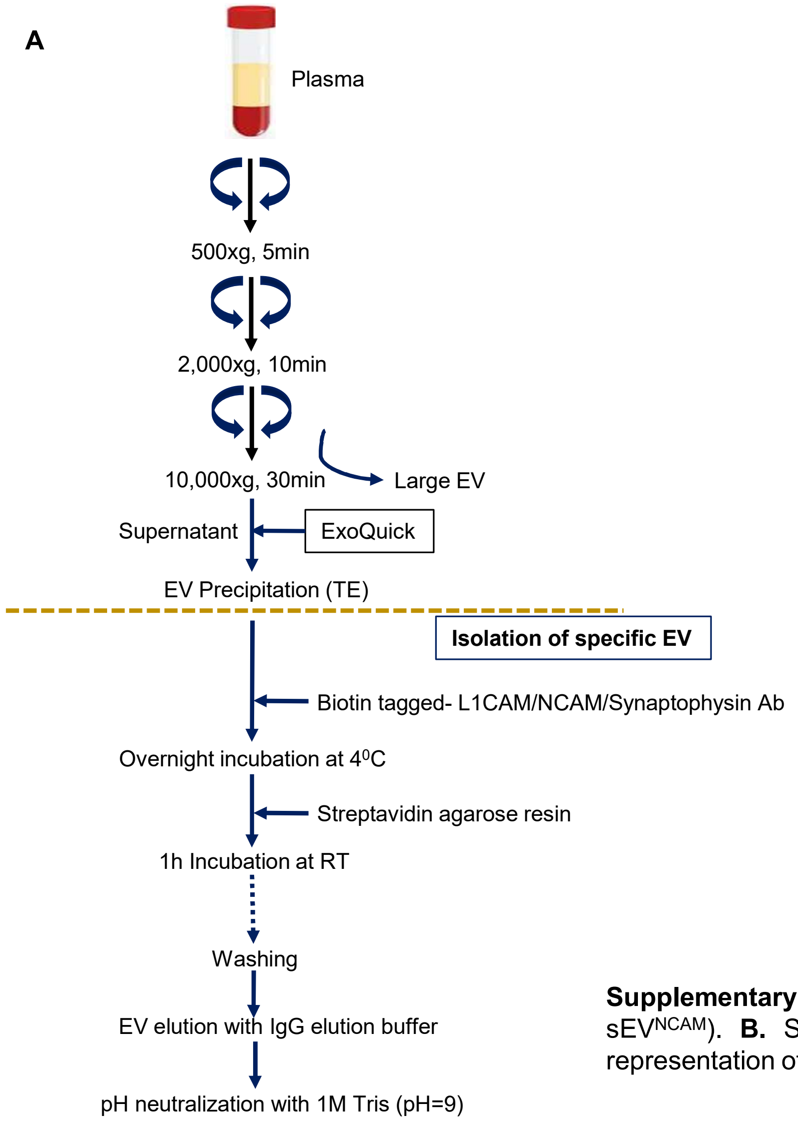

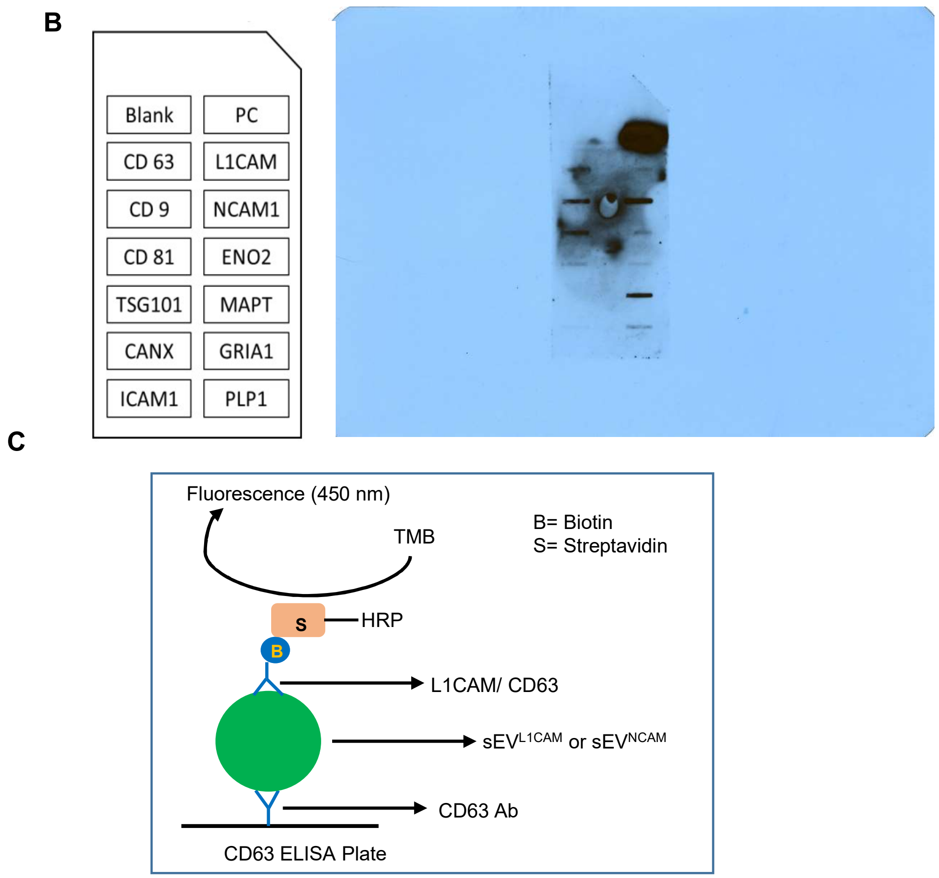

Supplementary Figure 1. A. Schematic diagram showing isolation of TE and NEE (sEV^{L1CAM}, sEV^{SYP} and sEV^{NCAM}). **B.** Scheme of Exo-Check (neuro) array and full blot of the array result. **C.** Schematic representation of experiment set up for confirmation of L1CAM co-expression with CD63 using ELISA.

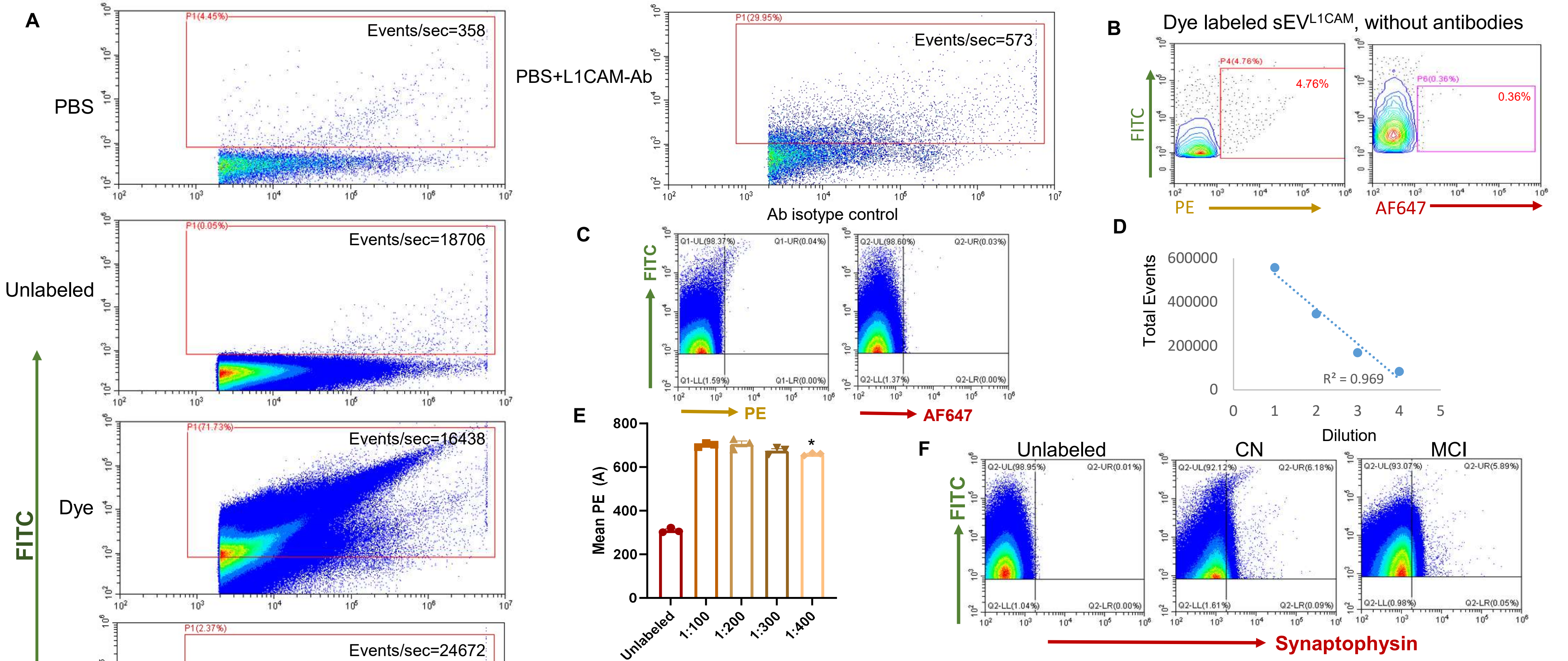
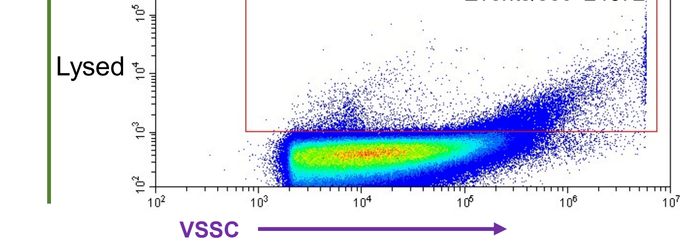

Supplementary Figure 2. A. PBS filtered through 0.22 micron filter was run to establish the background noise (upper panel). Threshold on VSSC channel was set at 2,000 to minimize the background noise. Total EV (TE) were acquired without labeling with CellBrite 488 dye and used to set the gate for positive events. TE after labeling with CellBrite 488 dye were captured for 60 sec. Particles in the gated regions were considered as EV. To confirm that the gated population is EV, 0.25% triton X-100 was added to lyse the EV and samples were acquired again. L1CAM-PE antibody in PBS was also run for 60 sec (upper right panel). **B.** EV labeled with dye but no antibody, was used to set the gate for antibody positive EV. **C.** Representative flow panels of Isotype antibody controls for PE and AF647 are shown. **D.** To further confirm detection of EV and negate any swarm effect, EV were diluted serially and acquired for 60 sec, dilution of EV showed the linear reduction in total events in the gated regions which confirmed that the positive events were EV. **E.** After co-labeling with CellBrite 488 dye and PE tagged L1CAM, serial dilution did not result in substantial reduction in the PE fluorescence in the gated population, while the fluorescence is much higher than the no antibody sample. Repeated measure of variance was used to analyze the statistical difference in PE fluorescence with different dilutions ($F= 6.03$, $df= (3,8)$. $^*p=0.011$). **F.** sEV^{SYP} were also estimated in TE using flow cytometry.

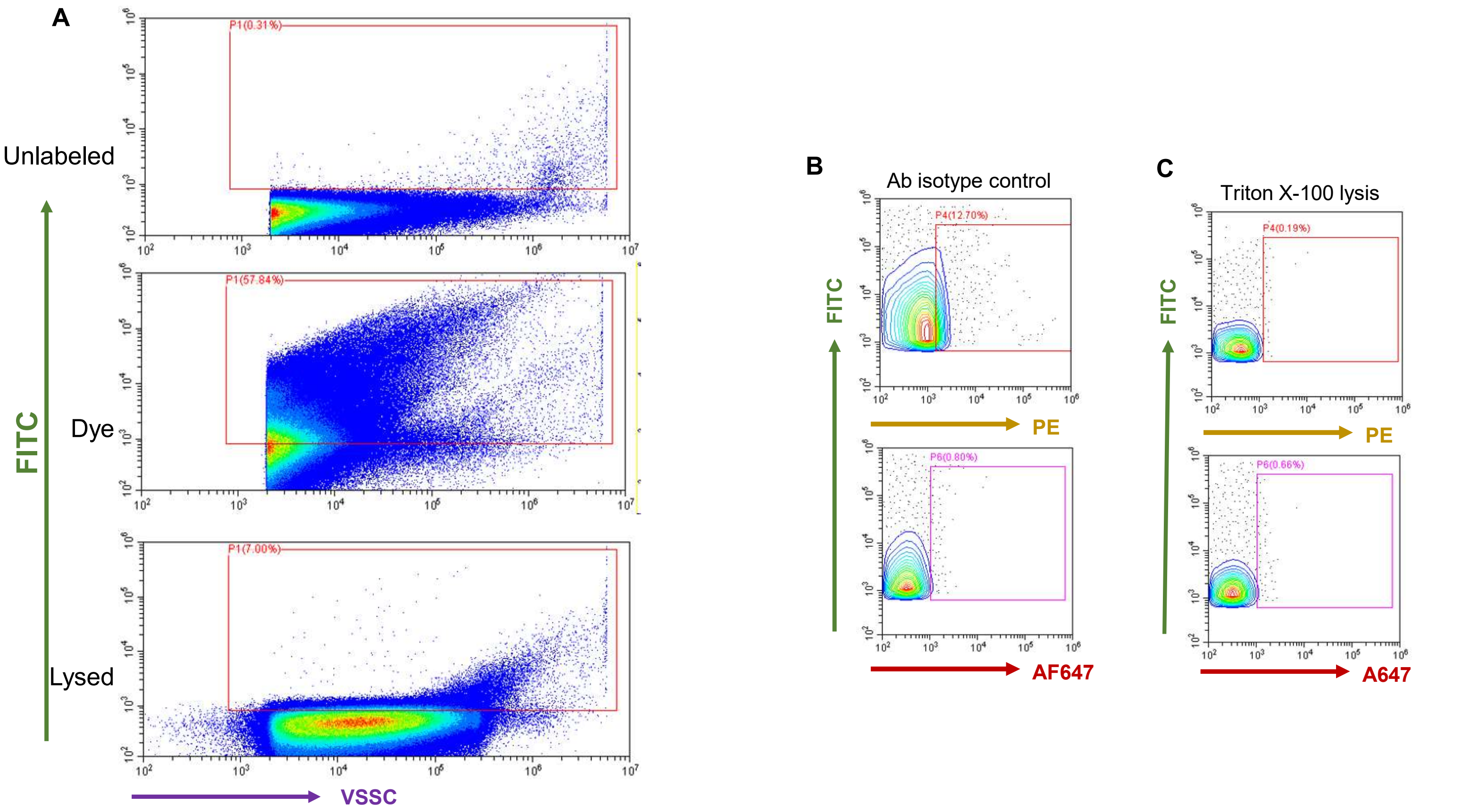

Supplementary Figure 3. A. Labeling of NEE was performed as discussed in supplementary Figure 2; NEE without dye (upper panel) was used to set up the gate for dye labeled NEE (middle panel). NEE were lysed using Triton X-100 for further confirmation (bottom panel). **B.** Both PE and AF647 labeled Isotype antibodies were used as control. **C.** NEE lysis completely abolished the fluorescence on PE and A647 labelled events confirming the capture of NEE.

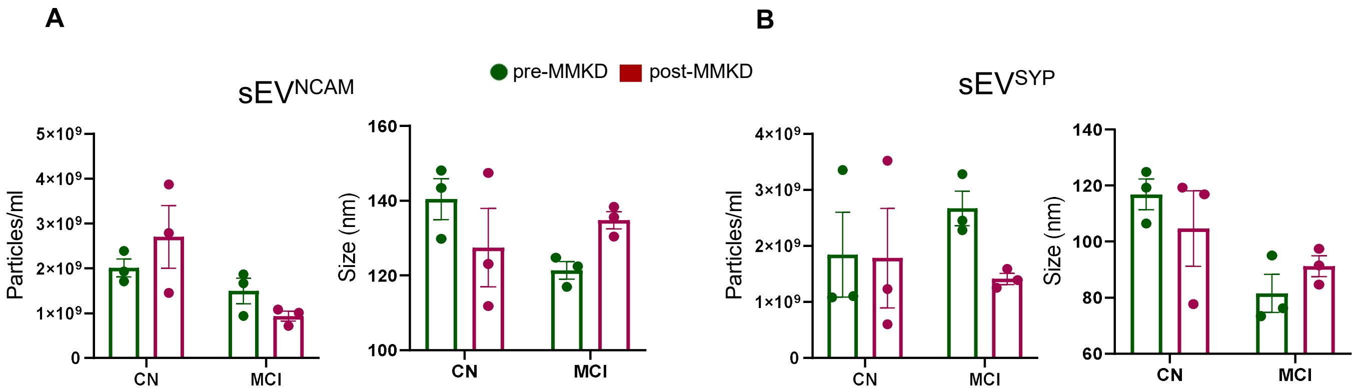

Supplementary Figure 4. A-B. Graphs represent the average concentration (left panel) and mean size (right panel) of sEV^{NCAM} and sEV^{SYP} in CN and MCI groups, pre and post MMKD. Error bars represents mean±SEM. Paired t-test was applied for comparing size, concentration and protein concentration. No statistically significant difference was observed.
