# Supplementary material for: Small extracellular vesicles in plasma reveal molecular effects of modified Mediterranean-ketogenic diet in participants with mild cognitive impairment
Source: Brain Commun. 2022 Oct 19;4(6):fcac262. doi: 10.1093/braincomms/fcac262 (PMC9629368; doi:10.1093/braincomms/fcac262)
Supplement: fcac262_Supplementary_Data [file fcac262_supplementary_data.zip › Supplementary_Tables.pdf]

Variable	All (n = 20)	CN (n = 11)	MCI (n = 9)
Sex (male/female)	5/15	2/9	3/6
APOE4 (±)	6/13	2/8	4/5
Age (y)	64.3 (6.3)	64.9 (7.9)	63.4 (4.0)
Education (y)	16.1 (2.5)	16.5 (2.3)	15.7 (2.9)
BMI (kg/m2)	28.4 (5.7)	26.9 (6.2)	30.3 (4.7)
MMSE (out of 30)	28.7 (1.1)	28.9 (1.0)	28.3 (1.2)
Glucose (mg/dL)	97.6 (18.3)	93.9 (11.4)	102.1 (24.4)
BHB (mmol/L)	0.23 (0.27)	0.35 (0.31)	0.1 (0.14)
Insulin (μIU/mL)	8.3 (6.1)	5.2 (3.4)	12.0 (6.8)
Hemoglobin A1c (%)	5.9 (0.3)	5.9 (0.2)	6.1 (0.4)
Total cholesterol (mg/dL)	215.2 (43.7)	200.5 (42.1)	233.2 (40.6)
HDL cholesterol (mg/dL)	67.4 (25.8)	69.5 (25.8)	64.8 (27.1)
VLDL cholesterol (mg/dL)	20.0 (11.1)	15.2 (6.2)	25.8 (13.2)
LDL cholesterol (mg/dL)	121.5 (41.2)	104.1 (45.6)	142.7 (24.2)
Triglycerides (mg/dL)	99.8 (55.7)	75.5 (30.5)	129.4 (66.4)

Supplementary Table 1. Description of the participants at baseline (means and standard deviations) included in the parent study to collect the plasma samples. (Abbreviations: BHB, β-hydroxybutyrate; BMI, body mass index; CN, Cognitively normal; MCI, mild cognitive impairment; MMSE, Mini-Mental State Examination; VLDL, very-low-density lipoprotein).

A.

Pearson Correlation Coefficients, N = 11 (CN)						
Prob > |r| under H0: Rho=0						
	Dif_MMKD_Aβ1-42	Dif_MMKD_NfL	Dif_MMKD_p181-tau	Dif_MMKD_t-tau	Dif_MMKD_Aβ1-42/p181-tau	Dif_MMKD_p181-tau/t-tau
Dif_MMKD_GRIN1	0.48242	0.18062	-0.25437	-0.29276	0.36431	0.24111
	0.1329	0.5951	0.4504	0.3823	0.2707	0.4751
Dif_MMKD_GRIA1	0.60136	-0.21536	-0.18624	-0.08674	0.50471	0.17110
	0.0503	0.5248	0.5835	0.7998	0.1133	0.6150
Dif_MMKD_GRIN2A	-0.21309	0.3047	-0.0489	-0.00394	-0.0954	0.04019
	0.5293	0.3623	0.8865	0.9908	0.7802	0.9066
Dif_MMKD_GRIN2B	-0.22284	0.24933	-0.08656	0.01894	-0.06161	0.04131
	0.5101	0.4597	0.8002	0.9559	0.8572	0.9040

B.

Pearson Correlation Coefficients, N = 9 (MCI)						
Prob > |r| under H0: Rho=0						
	Dif_MMKD_Aβ1-42	Dif_MMKD_NfL	Dif_MMKD_p181-tau	Dif_MMKD_t-tau	Dif_MMKD_Aβ1-42/p181-tau	Dif_MMKD_p181-tau/t-tau
Dif_MMKD_GRIN1	-0.87176	0.49527	-0.6063	0.80301	-0.38358	-0.05748
	0.0022	0.1752	0.0835	0.0092	0.3082	0.8832
Dif_MMKD_GRIA1	-0.22498	0.6441	0.40256	0.64269	-0.8433	-0.25756
	0.5606	0.0612	0.2827	0.0619	0.0043	0.5034
Dif_MMKD_GRIN2A	-0.17412	0.65518	0.48432	0.51858	-0.87558	-0.22064
	0.6541	0.0554	0.1864	0.1526	0.002	0.5684
Dif_MMKD_GRIN2B	-0.2518	0.72198	0.38657	0.58676	-0.88029	-0.27848
	0.5134	0.0281	0.3041	0.0967	0.0017	0.4681

Supplementary Table 2: Pearson correlation analysis of change in expression of different glutamate receptors with the change in levels of ADRD biomarkers in sEV^{L1CAM} following MMKD intervention in (**A)** CN (n=11) and (**B)** MCI (n=9) group. Pearson correlation coefficient (upper row) and statistical significance (lower row) are shown. Statistically significant values are highlighted in red ($p<0.05$). (Abbreviation: Dif, Difference)

A.

Pearson Correlation Coefficients, N = 11 (CN)						
Prob > |r| under H0: Rho=0						
	Dif_AHAD_Aβ1-42	Dif_AHAD_NfL	Dif_AHAD_p181-tau	Dif_AHAD_t-tau	Dif_AHAD_Aβ1-42/p181-tau	Dif_AHAD_p181-tau/t-tau
Dif_AHAD_GRIN1	-0.39904	0.76805	-0.51249	0.82851	-0.16185	-0.24883
	0.2241	0.0058	0.107	0.0016	0.6345	0.4606
Dif_AHAD_GRIA1	-0.50874	0.29526	-0.14654	0.51653	-0.42431	0.05617
	0.11	0.3781	0.6672	0.1038	0.1934	0.8697
Dif_AHAD_GRIN2A	0.49979	-0.38302	0.27423	-0.73074	0.36775	-0.08801
	0.1175	0.2449	0.4145	0.0106	0.2658	0.7969
Dif_AHAD_GRIN2B	0.4779	-0.3599	0.19513	-0.71944	0.37696	-0.11516
	0.1371	0.277	0.5653	0.0126	0.2531	0.7360

B.

Pearson Correlation Coefficients, N = 9 (MCI)						
Prob > |r| under H0: Rho=0						
	Dif_AHAD_Aβ1-42	Dif_AHAD_NfL	Dif_AHAD_p181-tau	Dif_AHAD_t-tau	Dif_AHAD_Aβ1-42/p181-tau	Dif_AHAD_p181-tau/t-tau
Dif_AHAD_GRIN1	0.30763	0.81805	-0.6435	0.90449	0.74335	-0.36985
	0.4206	0.007	0.0615	0.0008	0.0217	0.3272
Dif_AHAD_GRIA1	-0.02872	-0.79905	0.71006	-0.84388	-0.63619	0.34001
	0.9415	0.0098	0.0321	0.0042	0.0655	0.3706
Dif_AHAD_GRIN2A	0.39464	-0.36871	0.46682	-0.15041	0.0513	-0.01993
	0.2932	0.3288	0.2052	0.6993	0.8957	0.9594
Dif_AHAD_GRIN2B	0.23588	-0.18227	0.40867	-0.05258	-0.03766	-0.00944
	0.5412	0.6388	0.2748	0.8931	0.9234	0.9808

Supplementary Table 3: Pearson correlation analysis of change in expression of different glutamate receptors with the change in levels of AD biomarkers in sEV^{L1CAM} following AHAD intervention in **(A)** CN (n=11) and **(B)** MCI (n=9) group. Pearson correlation coefficient (upper row) and statistical significance (lower row) are shown. Statistically significant values are highlighted in red ($p<0.05$). (Abbreviation: Dif, Difference)
